# Long-Term Prognostic Value of AFP and PIVKA-II in HCC After Living Donor Liver Transplantation: A Single-Center Retrospective Study

**DOI:** 10.3389/ti.2025.14748

**Published:** 2025-06-27

**Authors:** Saran Ochir Gongor, YoungRok Choi, Gayoung Kim, Min Kyoung Kim, Sang Hyuk Park, Jiyoung Kim, Jae-Yoon Kim, Su young Hong, Jeong-Moo Lee, Suk Kyun Hong, Kwang-Woong Lee

**Affiliations:** ^1^ Department of Surgery, Seoul National University College of Medicine, Seoul, Republic of Korea; ^2^ Department of Surgery, Seoul National University Hospital, Seoul, Republic of Korea

**Keywords:** hepatocellular carcinoma (HCC), alpha-fetoprotein, living donor liver transplantation (LDLT), des-gamma carboxyprothrombin, tumor biomarker, PIVKA-II

## Abstract

Despite the development of numerous prognostic models for hepatocellular carcinoma (HCC) recurrence and mortality after liver transplantation, tumor biomarkers such as alpha-fetoprotein (AFP) and protein induced by vitamin K absence-II (PIVKA-II) remain widely used in clinical practice. This study evaluated the performance of AFP and PIVKA-II compared with six prognostic models (RETREAT, SNAPP, MoRAL, R3-AFP, METROTICKET 2.0, and SALT) in a retrospective cohort of 707 adults who underwent living donor liver transplantation (LDLT) for HCC between 2003 and 2018. Patients were stratified into Milan and Beyond Milan groups. Time-dependent receiver operating characteristic curve analysis was conducted using integrated area under the curve (iAUC) and concordance index (C-index) to assess recurrence and mortality. AFP and PIVKA-II (continuous) achieved iAUCs of 0.68–0.75 for recurrence and C-indices of 0.66–0.77 for mortality. Their combination reached iAUCs up to 0.78 and C-indices up to 0.80. Threshold models (AFP ≥200, PIVKA-II ≥400) showed modest predictive performance. Among multivariable models, R3-AFP demonstrated the most consistent performance (iAUC 0.76–0.81; C-index 0.78–0.82). SNAPP, MoRAL, and SALT also performed well. AFP and PIVKA-II may offer practical utility in resource-limited settings. However, multivariable models remain the preferred approach where comprehensive diagnostics are available.

## Graphical Abstract

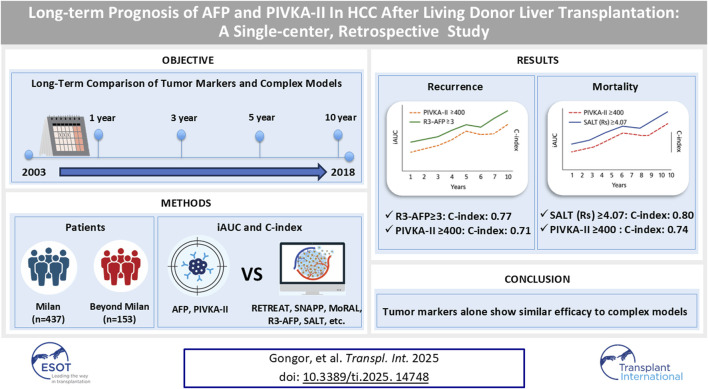

## Introduction

Hepatocellular carcinoma (HCC) recurrence remains a concern in liver transplantation (LT), with rates ranging from 10% to 21% [1–6]. Post-LT recurrence also significantly contributes to patient mortality [2, 4, 6], despite advances in surgical and perioperative care. The Milan criteria have historically ensured excellent post-LT outcomes [2, 3, 5]. However, with an increasing number of recipients exceeding these strict morphological boundaries, the Milan criteria alone are no longer sufficient to accurately predict post-transplant outcomes.

Owing to these limitations, particularly their emphasis on tumor size and number, a paradigm shift has emerged toward incorporating tumor biology using surrogate tumor biological markers [2, 7]. This approach aims to better capture the intrinsic behavior of HCC and enhance the selection process for LT candidates [2, 7]. Moreover, the lack of standardized post-LT HCC surveillance guidelines [8, 9] has prompted the development of diverse prognostic scoring systems and refined selection protocols [3, 5, 10]. These models now integrate a broader range of factors, including radiological [2], molecular [2], serological [2, 11], and morphological [2, 5, 9] factors to promote recurrence detection and improve post-transplant survival.

The rising demand for living donor liver transplantation (LDLT) [12, 13], which frequently involves patients beyond the Milan criteria, further highlights the need for expanded selection criteria and LDLT-specific predictive models [10, 12, 13]. Consequently, several prognostic systems for LDLT have been developed, particularly in Asian centers [12, 13]. In 2016, our center introduced the Model for Recurrence After Liver Transplantation (MoRAL), a prognostic score based solely on surrogate tumor biological markers, which showed strong predictive performance, but still required complex calculations [14]. Similarly, another major Korean center proposed the SNAPP (Size and Number, alpha-fetoprotein (AFP), protein induced by vitamin K absence-II (PIVKA-II), and positron-emission tomography (PET) score, which incorporates morphological, biological (AFP and PIVKA-II), and radiological (PET) factors, demonstrating excellent prognostic utility, but requires PET results [15].

Moreover, in Western countries, the majority of LT is performed with deceased donor liver transplantation (DDLT) settings; therefore, most prognostic systems are developed based on DDLT. This could be another reason for the unlimited access to certain LDLT centers [14–17]. In particular, among deceased-donor LT (DDLT) cohorts, scoring systems such as the Risk Estimation of Tumor Recurrence After Transplant (RETREAT), which integrates additional microvascular invasion status [16], and the recurrence-risk reassessment AFP (R3-AFP) [17], have also been developed and have demonstrated strong predictive performance for HCC recurrence but still require additional factors such as histopathologic differentiation grade. For survival-specific outcomes, tools such as the Survival After Liver Transplantation (SALT) calculator [18] and METROTICKET 2.0 [19] have yielded concordance (C)-indices exceeding 70%. It is fair to say that most high-volume centers worldwide proposed their own selection criteria or scoring systems for HCC in LT; however, unlike during the Milan era, these models are not uniformly reached to an consensus or consistently adopted in routine clinical practice [2, 7]. Since the target groups differ [14–17], their actual clinical applicability is limited. Moreover, despite the robust predictive performance of these models, their complexity and reliance on advanced diagnostics [8, 20] hamper their routine clinical implementation. Consequently, only a few of these scoring systems are consistently utilized in clinical practice, especially in LDLT [8, 20].

Advances in surgical techniques, immunosuppressive strategies, and systemic therapies, including immune checkpoint inhibitors [21], have significantly improved long-term post-transplant survival [22], with mean survival rates now exceeding 20 years [23, 24]. Despite this progress, long-term outcomes, such as HCC recurrence and mortality over extended periods, remain insufficiently understood [25]. Most prognostic models are designed to predict recurrence or post-LT mortality within 3 or 5 years [2, 7]. In particular, there is a lack of validated, simplified prognostic models for long-term (>5 years) outcomes after LDLT that rely solely on biochemical markers such as AFP and PIVKA-II [25].

In this context, AFP [1, 11, 26–30] and PIVKA-II [26, 31] levels remain pivotal as readily obtainable biomarkers, with decades-long validation as accurate and reliable indicators in clinical practice, and continue to provide crucial insights into tumor recurrence and survival outcomes through simple and singular measurements. More complex prognostic models, however, require additional measurements, and their use is generally limited to specific circumstances [17, 20], meaning they are typically only available in tertiary or quaternary medical facilities.

We hypothesized that AFP and PIVKA-II levels would show performance comparable to that of highly accurate complex prognostic models, particularly for predicting recurrence, mortality, and outcomes beyond 5-year post-LT, while being simpler to use. This study, therefore, compared the predictive accuracy of models for predicting recurrence and mortality with that of these two traditional biomarkers. Additionally, we evaluated the accuracy of the predictive ability of individual and combined values of AFP and PIVKA-II for predicting outcomes beyond 5-year post-LT and their utility in predicting location-specific HCC recurrence.

## Materials and Methods

### Study Design and Patients

**FIGURE 1:**
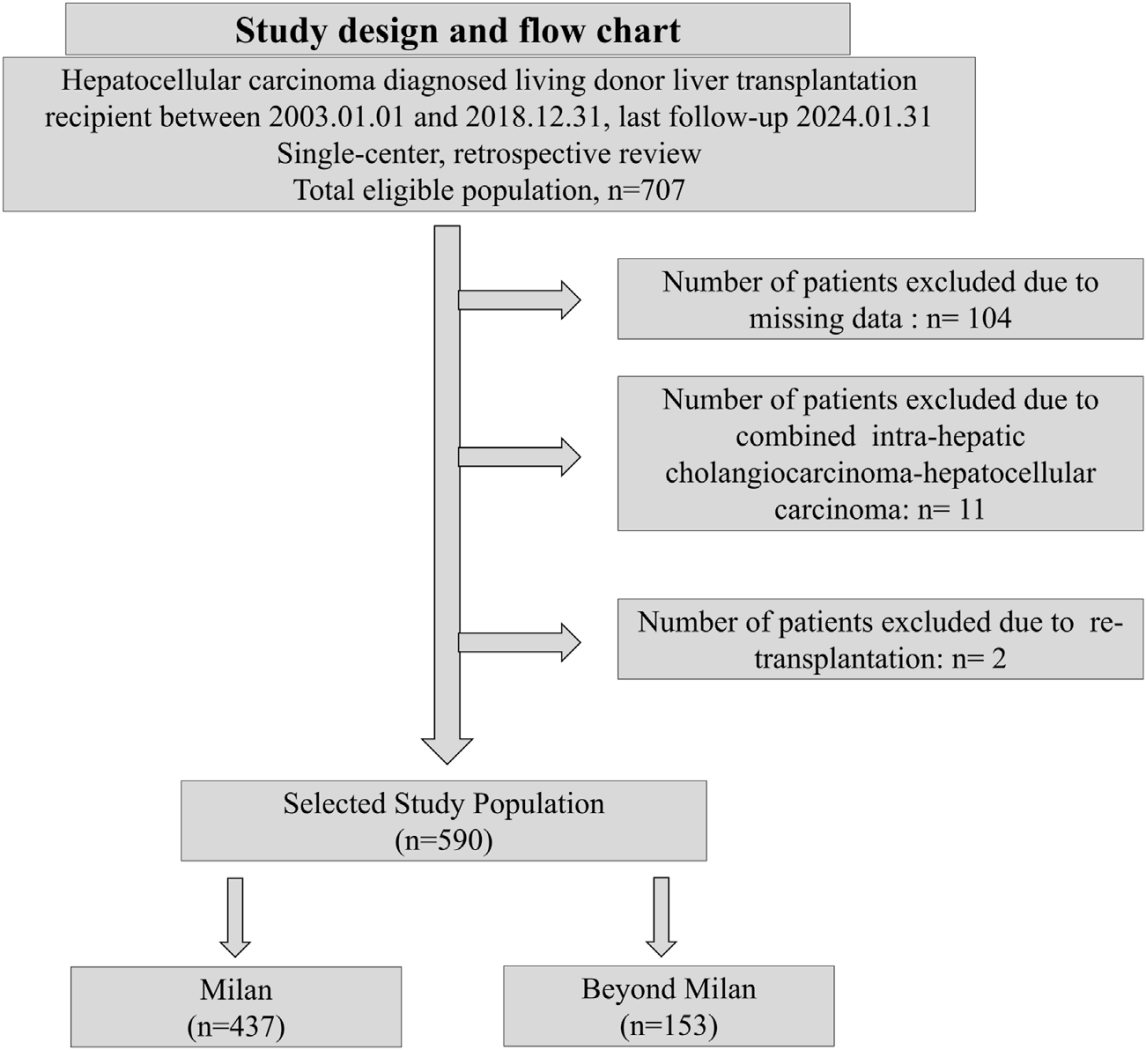
Study design and flow chart.

A single-center, retrospective analysis was performed on 707 patients with HCC who underwent adult LDLT at Seoul National University Hospital between 2003.01.01 and 2018.12.31. The last follow-up date was 2024.01.31 (Figure 1). The inclusion criteria were as follows: 1. age ≥19 years; 2. diagnosis of HCC following LDLT based on explant pathology reports; and 3. with available AFP and PIVKA-II measurements prior to transplantation. The exclusion criteria were as follows: 1. non-diagnosis of HCC following LDLT based on explant pathology; 2. re-transplantation; 3. missing AFP and PIVKA-II measurements; and 4. combined intra-hepatic carcinoma-hepatocellular carcinoma or intra-hepatic carcinoma. Diagnosis of HCC was based on postoperative histopathological examination. The study patients were subsequently categorized into the Milan cohort (MC) and Beyond Milan cohort (BMC) based on explant pathology.

### Data Collection

Preoperative data, including demographic and clinical information, such as age, sex, body mass index (BMI), arterial hypertension, diabetes mellitus, underlying disease (such as HCV and HBV infections), AFP level, PIVKA-II level, and Model for End-stage Liver Disease scores, were extracted from electronic medical records (EMRs). Records of pre-transplant interventions, such as hepatectomy, transarterial chemoembolization, radiofrequency ablation, and percutaneous ethanol injection therapy, were reviewed from the EMRs. Tumor characteristics, including vascular invasion status (microvascular or macrovascular), tumor size, number of tumors, tumor stage, and differentiation grade, were extracted from post-LT pathological reports. HCC diagnosis, total necrosis, and graft-to-recipient weight ratio were confirmed by explant pathology findings. Overall survival (OS) was defined as the period from the date of liver transplantation until death from any cause. Patients who were alive at the last follow-up were censored. Recurrence-free survival (RFS) was defined as the duration from the date of liver transplantation to the first radiological or pathological evidence of HCC recurrence or death from any cause, whichever occurred first. Patients without recurrence or death were censored at the last follow-up date.

### Assessment of Cox Prediction Models and Statistical Analysis

Preoperative AFP and PIVKA-II levels, as individual predictors, and four prognostic scores for HCC recurrence (SNAPP, RETREAT, MoRAL, and R3-AFP) were evaluated in both the MC and BMC [14–17]. AFP, PIVKA-II, SALT, and METROTICKET 2.0 scores were evaluated to predict post-LT mortality for both the MC and BMC [18, 19]. All prognostic scores were calculated retrospectively utilizing explant pathology reports, preoperative measurements, and radiological data. Time-dependent receiver operating characteristic (ROC) analysis was performed using Uno’s integrated area under the curve (iAUC), with inverse probability of censoring weighting (IPCW), to evaluate the dynamic predictive performance of the Cox regression models applied to both composite scores and individual biological tumor markers for HCC recurrence and mortality [32]. Furthermore, to assess the overall predictive ability of each model, Harrell’s C-index was calculated from the Cox regression models for both recurrence and mortality outcomes [33]. Confidence intervals for Uno’s iAUC were derived from 1,000 bootstrap samples, and iAUC values were compared between models using 1,000 bootstrap iterations to ensure the robustness and statistical reliability of the estimate [34, 35].

Continuous variable Cox regression models utilized calculated scores from prognostic models or measurements of biological markers, such as AFP and PIVKA-II levels, to predict HCC recurrence or survival following LDLT. In contrast, the threshold value binary Cox regression models were constructed using previously reported or validated threshold values (Supplementary Table S3). The Cox model specifications were as follows: AFP ≥200 (ng/mL) and PIVKA-II ≥400 (mAU/mL) cutoff values for predicting HCC recurrence and mortality were employed, as previously described [36, 37]. The SNAPP score was calculated using AFP, PIVKA-II, tumor size, number, and PET metabolic status, with a SNAPP ≥5 score used as a cutoff to indicate a high risk of HCC recurrence, as previously described [15]. The RETREAT score was calculated using the explant microvascular invasion status, largest tumor size, and preoperative AFP level, with RETREAT ≥5 indicating the high-risk group and as a threshold, as previously described [16]. The MoRAL score was calculated using preoperative AFP and PIVKA-II levels with a cutoff of 314.8, as previously described [14]. The R3-AFP score was based on the number of nodules, size of the largest tumor nodule, AFP level, microvascular invasion status, and tumor differentiation grade (Edmonson and Steiner grade >2), with a cutoff R3-AFP ≥3 score for cox models, as previously described [17]. SALT was based on the risk score, with a risk score ≥4.07 used as a threshold for higher mortality, as previously described [18]. The METROTICKET transplantability score was based on three categories: 1. If AFP <200 ng/mL, the sum of the number and size ≤7; 2; if 200 ≤ AFP <400 ng/mL, the sum of the number and size ≤5; 3. if 400 ≤ AFP <1,000 ng/mL, the sum of the number and size ≤4, as described previously [19].

The Kaplan–Meier method was used to estimate overall survival and recurrence-free survival. Chi-square or Fisher’s exact tests were used to compare categorical variables, and Student’s t-test was used for continuous variables. Statistical analyses were performed using SPSS version 29 (IBM SPSS Inc., Armonk, NY, United States) and R version 4.4.1^1^. Uno’s iAUC was calculated using “survAUC” package, and Harrell’s C-index was computed using “survcomp” package in R. Cox models were fitted using “survival.” All statistical tests were two-sided, with a significance threshold of 0·05, and were performed within an exploratory framework.

### Ethical Statements

This study adhered to the Declaration of Helsinki and Istanbul guidelines and was approved by the Institutional Review Board of Seoul National University Hospital (IRB-H-2502-060-1612). The need to obtain informed patient consent was waived owing to the retrospective nature of the study. This study adhered to the STROBE guideline for retrospective study and check lists are provided in the Supplementary Material.

## Results

### Baseline Characteristics and Post-LT Outcomes

From 707 screened patients, 117 were excluded due to missing data, combined cholangiocarcinoma-hepatocellular carcinoma, or re-transplantation history. Finally, 590 patients who underwent LDLT were categorized into the MC (n = 437) and BMC (n = 153) groups (Figure 1). The baseline characteristics were similar between the groups (Table 1). Hepatitis B was more common in the MC (81.0% vs. 75.8%), whereas hepatitis C was more common in the BMC (13.1% vs. 8.2%, p < 0.01). BMC showed elevated tumor markers with higher AFP (15,125.0 vs. 48.4 ng/mL, p = 0.03) and PIVKA-II levels (3,556.6 vs. 69.4 mAU/mL, p < 0.01). More BMC patients had AFP ≥200 ng/mL and PIVKA-II ≥400 mAU/mL. Prognostic scores, tumor characteristics, and PET-CT hypermetabolic activity were worse in the BMC (44.4% vs. 11.7%, p < 0.01, Table 1; Supplementary Table S1). BMC patients had higher recurrence rates (50.3% vs. 10.3%), mortality (46.4% vs. 15.6%), and HCC-specific deaths (87.3% vs. 45.6%, Table 2). The median follow-up was longer in the MC group (113.05 vs. 70.54 months, Table 2), whereas recurrence-free survival was shorter in the BMC group (51.8 vs. 89.8 months, p < 0.01). The median follow-up duration for the entire population was 104.6 months (IQR: 68.0–145.9 months).

**TABLE 1:** Baseline characteristics of study population.

Variables	Milan Cohort (N = 437)	Beyond Milan Cohort (N = 153)	p-value
Age (years)	55.5 ± 7.5	56.0 ± 9.4	0.52
Sex, male	359 (82.2%)	137 (89.5%)	0.03
BMI	24.2 ± 11.9	23.5 ± 3.3	0.49
Hypertension	44 (10.1%)	16 (10.5%)	0.89
Diabetes mellitus	70 (17.6%)	24 (15.7%)	0.59
MELD score	13.4 ± 7.1	13.1 ± 6.5	0.66
Underlying disease			0.15
HBV	354 (81.0%)	116 (75.8%)	
HCV	36 (8.2%)	20 (13.1%)	
Alcoholic liver disease	29 (6.6%)	7 (4.6%)	
Others	18 (4.1%)	10 (6.5%)	
AFP (ng/mL)	48.4 ± 149.9	15,125.0 ± 140,747.1	0.03
AFP ≥200 (ng/mL)	26 (5.9%)	32 (20.9%)	<0.01
PIVKA-II (mAU/mL)	69.5 ± 155.4	3556.6 ± 11,703.0	<0.01
PIVKA-II ≥400 (mAU/mL)	16 (3.7%)	38 (24.8%)	<0.01
MoRAL score	81.54 ± 58.51	359.74 ± 687.84	<0.01
MoRAL ≥314.8	5 (1.1%)	35 (22.9%)	<0.01
RETREAT score	1.4 ± 1.0	3.2 ± 2.0	<0.01
RETREAT ≥5	6 (1.4%)	38 (24.8%)	<0.01
SNAPP score	0.9 ± 1.0	3.6 ± 1.7	<0.01
SNAPP ≥5	3 (0.7%)	41 (26.8%)	<0.01
Risk score (SALT)	2.3 ± 0.6	3.4 ± 1.3	<0.01
Risk score (SALT) ≥4.07	4 (0.9%)	41 (26.8%)	<0.01
R3-AFP score	0.8 ± 0.9	3.7 ± 2.4	<0.01
R3-AFP ≥3	18 (4.1%)	88 (57.5%)	<0.01
METROTICKET 2.0 transplantability			<0.01
Eligible	416 (95.2%)	51 (33.3%)	
Ineligible	21 (4.8%)	102 (66.7%)	

Values are present with number (%) or mean ±SD or median (IQR).

BMI, body mass index; MELD, Model for End-Stage Liver Disease; HBV, hepatitis B virus; HCV, hepatitis C virus; LT, liver transplantation; TACE, transarterial chemoembolization; RFA, radiofrequency ablation; PEIT, percutaneous ethanol injection therapy; AFP, alpha-fetoprotein; PIVKA-II, protein induced by vitamin K absence-II; MoRAL, Model for Recurrence After Liver Transplantation; RETREAT, Risk Estimation of Tumor Recurrence After Transplant; SNAPP, Size and Number, AFP, PIVKA-II, PET; SALT, Survival After Liver Transplantation; R3-AFP, Recurrence-Risk Reassessment AFP.

**TABLE 2:** Post-LT outcomes.

Variables	Milan Cohort (N = 437)	Beyond Milan Cohort (N = 153)	p-value
Recurrence	45 (10.3%)	77 (50.3%)	<0.01
Intra-hepatic	10 (2.3%)	22 (14.4%)	<0.01
Extra-hepatic	35 (8.0%)	55 (35.9%)	<0.01
Number of intra-hepatic recurred tumor			0.77
1	6 (50.0%)	17 (47.2%)	
2	1 (8.3%)	6 (16.7%)	
Multiple	5 (41.7%)	13 (36.1%)	
Post-Recurrence Treatment			0.24
Untreated	6 (13.3%)	5 (6.5%)	
Transarterial chemoembolization	10 (22.2%)	28 (36.4%)	
Surgical resection	18 (40.0%)	21 (27.3%)	
Radiotherapy	6 (13.3%)	18 (18.2%)	
Chemotherapy	3 (6.7%)	8 (10.4%)	
Radiofrequency ablation	2 (4.4%)	1 (1.3%)	
Mortality	68 (15.6%)	71 (46.4%)	<0.01
HCC specific mortality	31 (45.6%)	62 (87.3%)	<0.01
Median follow-up, months (IQR)	113.1 (77.7–149.0)	70.5 (27.3–135.8)	<0.01
Median recurrence free survival, months (IQR)	89.8 ± 56.4	51.8 (11.8–128.2)	<0.01

Values are present with number (%) or mean ±SD or median (IQR) HCC, hepatocellular carcinoma; IQR, interquartile range.

### Post-LT HCC Recurrence and Mortality Prediction

**FIGURE 2:**
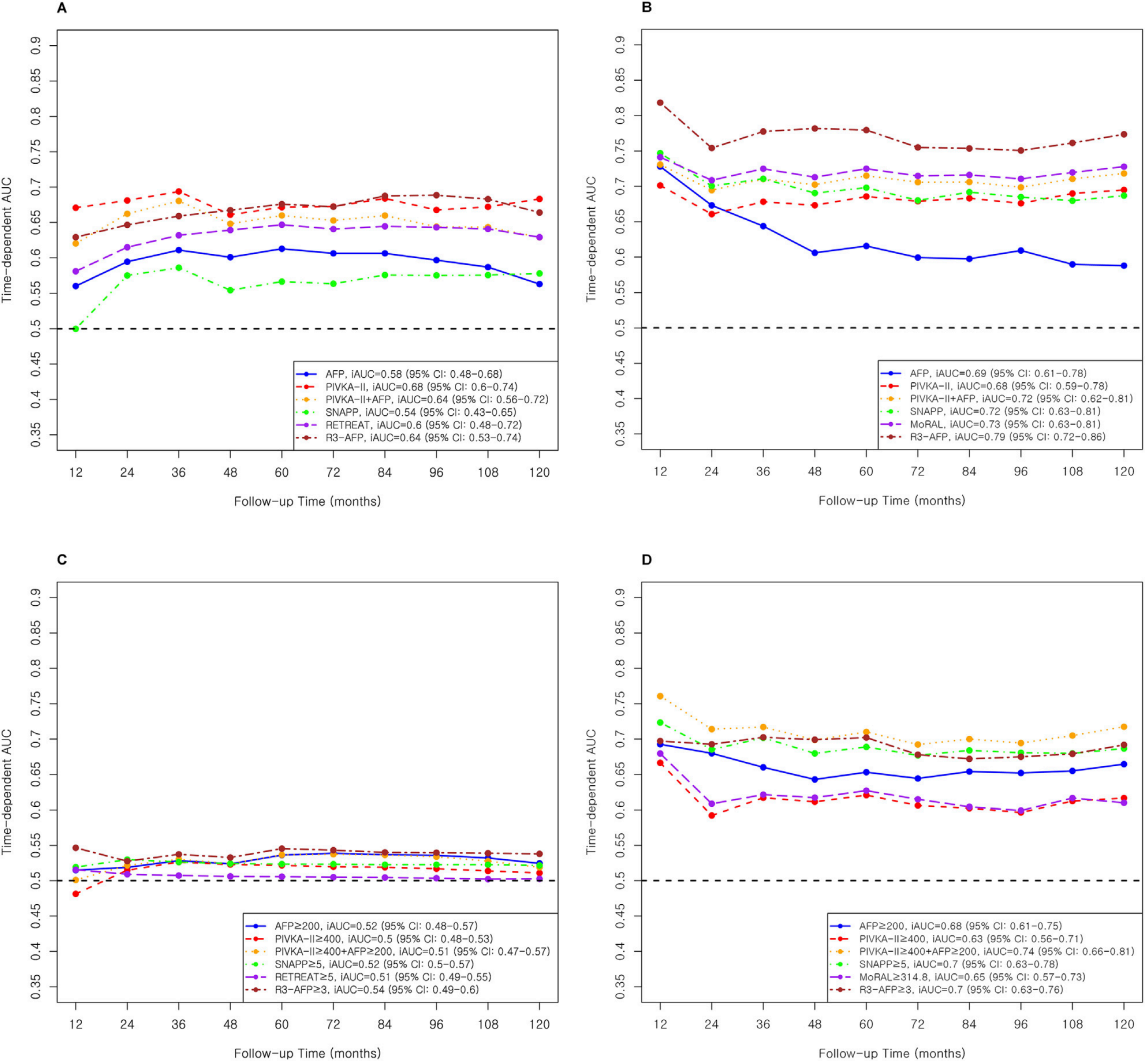
AUC and iAUC for Recurrence Prediction in Milan and Beyond Milan cohort. **(A)** Continuous variable–based AUC and iAUC in the Milan cohort. **(B)** Continuous variable–based AUC and iAUC in the Beyond Milan cohort. **(C)** Threshold-based AUC and iAUC in the Milan cohort. **(D)** Threshold-based AUC and iAUC in the Beyond Milan cohort.

Individual tumor markers showed modest predictive performance (Table 3; Figure 2). In the Milan cohort, AFP (continuous, Table 3; Figure 2A) yielded an iAUC of 0.58 (95% CI: 0.47–0.68) and PIVKA-II 0.68 (95% CI: 0.60–0.75). In the Beyond Milan cohort, AFP and PIVKA-II achieved iAUCs of 0.69 (95% CI: 0.61–0.79) and 0.68 (95% CI: 0.58–0.78, Table 3; Figure 2B), respectively. Threshold models followed similar trends. AFP ≥200 had C-indices of 0.71 (95% CI: 0.53–0.90) in Milan and 0.76 (95% CI: 0.66–0.86) in Beyond Milan; PIVKA-II ≥400 reached 0.73 (95% CI: 0.52–0.93) and 0.71 (95% CI: 0.60–0.83). Combination marker models (AFP + PIVKA-II) showed improved performance. The continuous model yielded iAUCs of 0.64 (95% CI: 0.55–0.72) in Milan and 0.72 (95% CI: 0.62–0.80) in Beyond Milan. The threshold version yielded C-indices of 0.68 (95% CI: 0.50–0.86) and 0.73 (95% CI: 0.66–0.81). The MoRAL model, designed for Beyond Milan populations, achieved an iAUC of 0.65 (95% CI: 0.58–0.73, Figure 2D) and a C-index of 0.75 (95% CI: 0.65–0.86). Models incorporating morphology and vascular invasion showed stronger performance. R3-AFP (continuous, Table 3; Figure 2A) yielded iAUCs of 0.64 (95% CI: 0.54–0.74, Figure 2A) in Milan and 0.79 (95% CI: 0.71–0.86, Figure 2B) in Beyond Milan. R3-AFP ≥3 showed C-indices of 0.76 (95% CI: 0.58–0.93) and 0.77 (95% CI: 0.67–0.87). RETREAT, evaluated specifically in the Milan cohort, showed an iAUC of 0.60 (95% CI: 0.48–0.72), and RETREAT ≥5 achieved a C-index of 0.75 (95% CI: 0.30–1.00). The SNAPP model, which incorporates tumor biology, morphology, vascular invasion, and PET metabolism, showed an iAUC of 0.54 (95% CI: 0.42–0.65) in Milan and 0.72 (95% CI: 0.63–0.82) in Beyond Milan. SNAPP ≥5 demonstrated (Table 3; Figure 2) the strongest threshold performance: 0.88 (95% CI: 0.72–1.00) in Milan and 0.78 (95% CI: 0.69–0.87) in Beyond Milan.

**TABLE 3:** Integrated AUC and C-index of HCC recurrence of both Milan and Beyond Milan cohorts.

Prediction Cox Models	Milan Cohort (N = 437)		Beyond Milan Cohort (N = 153)	
	Recurrence		Recurrence	
	iAUC (95% CI)	C-index (95% CI)	iAUC (95% CI)	C-index (95% CI)
AFP (continuous)	0.58 (0.47–0.68)	0.64 (0.56–0.72)	0.69 (0.61–0.79)	0.63 (0.57–0.68)
AFP ≥200 (threshold)	0.52 (0.48–0.57)	0.71 (0.53–0.9)	0.68 (0.61–0.75)	0.76 (0.66–0.86)
PIVKA-II (continuous)	0.68 (0.6–0.75)	0.64 (0.57–0.72)	0.68 (0.58–0.78)	0.62 (0.55–0.69)
PIVKA-II ≥400 (threshold)	0.5 (0.48–0.53)	0.73 (0.52–0.93)	0.63 (0.56–0.78)	0.71 (0.6–0.83)
PIVKA-II + AFP (continuous)	0.64 (0.55–0.72)	0.66 (0.58–0.73)	0.72 (0.62–0.8)	0.64 (0.58–0.71)
PIVKA-II ≥400+AFP ≥200 (threshold)	0.51 (0.46–0.57)	0.68 (0.5–0.86)	0.74 (0.65–0.82)	0.73 (0.66–0.81)
SNAPP (continuous)	0.54 (0.42–0.65)	0.57 (0.45–0.7)	0.72 (0.63–0.82)	0.68 (0.61–0.76)
SNAPP ≥5 (threshold)	0.52 (0.5–0.56)	0.88 (0.72–1)	0.73 (0.65–0.81)	0.78 (0.69–0.87)
RETREAT (continuous)	0.6 (0.48–0.72)	0.69 (0.58–0.8)		
RETREAT ≥5 (threshold)	0.51 (0.49–0.55)	0.75 (0.3–1)		
MoRAL (continuous)			0.65 (0.58–0.73)	0.65 (0.59–0.72)
MoRAL ≥314.8 (threshold)			0.65 (0.58–0.73)	0.75 (0.65–0.86)
R3-AFP (continuous)	0.64 (0.54–0.74)	0.74 (0.64–0.83)	0.79 (0.71–0.86)	0.71 (0.64–0.77)
R3-AFP ≥3 (threshold)	0.54 (0.48–0.6)	0.76 (0.58–0.93)	0.7 (0.63–0.76)	0.77 (0.67–0.87)

iAUC, integrated area under curve; C-index, concordance index; 95%CI, 95% confidence interval; AFP, alpha-fetoprotein; PIVKA-II, protein induced by vitamin K absence-II; SNAPP, size and number, AFP, PIVKA-II, PET; RETREAT, risk estimation of tumor recurrence after transplant; MoRAL, model for recurrence after liver transplantation; R3-AFP, Recurrence-Risk Reassessment AFP.

**FIGURE 3:**
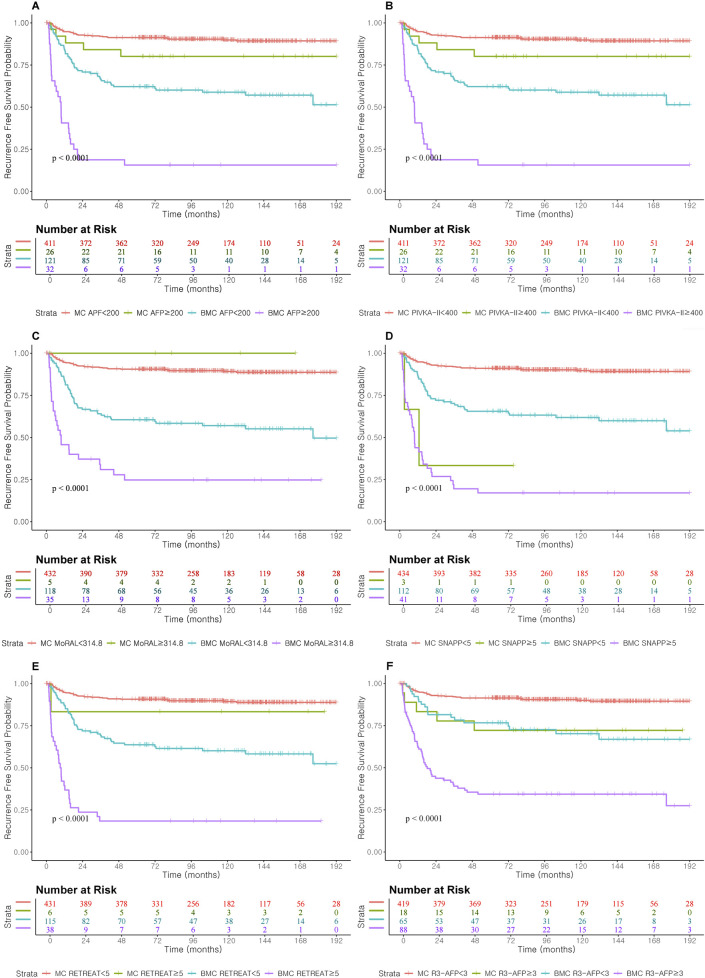
Recurrence Free Survival according to prognostic score cut-off and AFP and PIVKA-II cut-off level in Milan and Beyond Milan cohort. **(A)** Recurrence Free Survival according to AFP ≥200. **(B)** Recurrence Free Survival according to PIVKA-II ≥400. **(C)** Recurrence Free Survival according to MORAL ≥314.8. **(D)** Recurrence Free Survival according to SNAPP ≥5. **(E)** Recurrence Free Survival according to RETREAT ≥5. **(F)** Recurrence Free Survival according to R3-AFP ≥3.

Individual tumor markers, including AFP and PIVKA-II, showed moderate predictive accuracy, with slightly better performance in the Beyond Milan cohort (Table 3). While combining these markers modestly improved discrimination, complex models such as R3-AFP and SNAPP ≥5 consistently showed higher iAUCs and C-indices across both cohorts. Recurrence-free survival for each threshold model and marker was evaluated, and results are shown in Figure 3.

**FIGURE 4:**
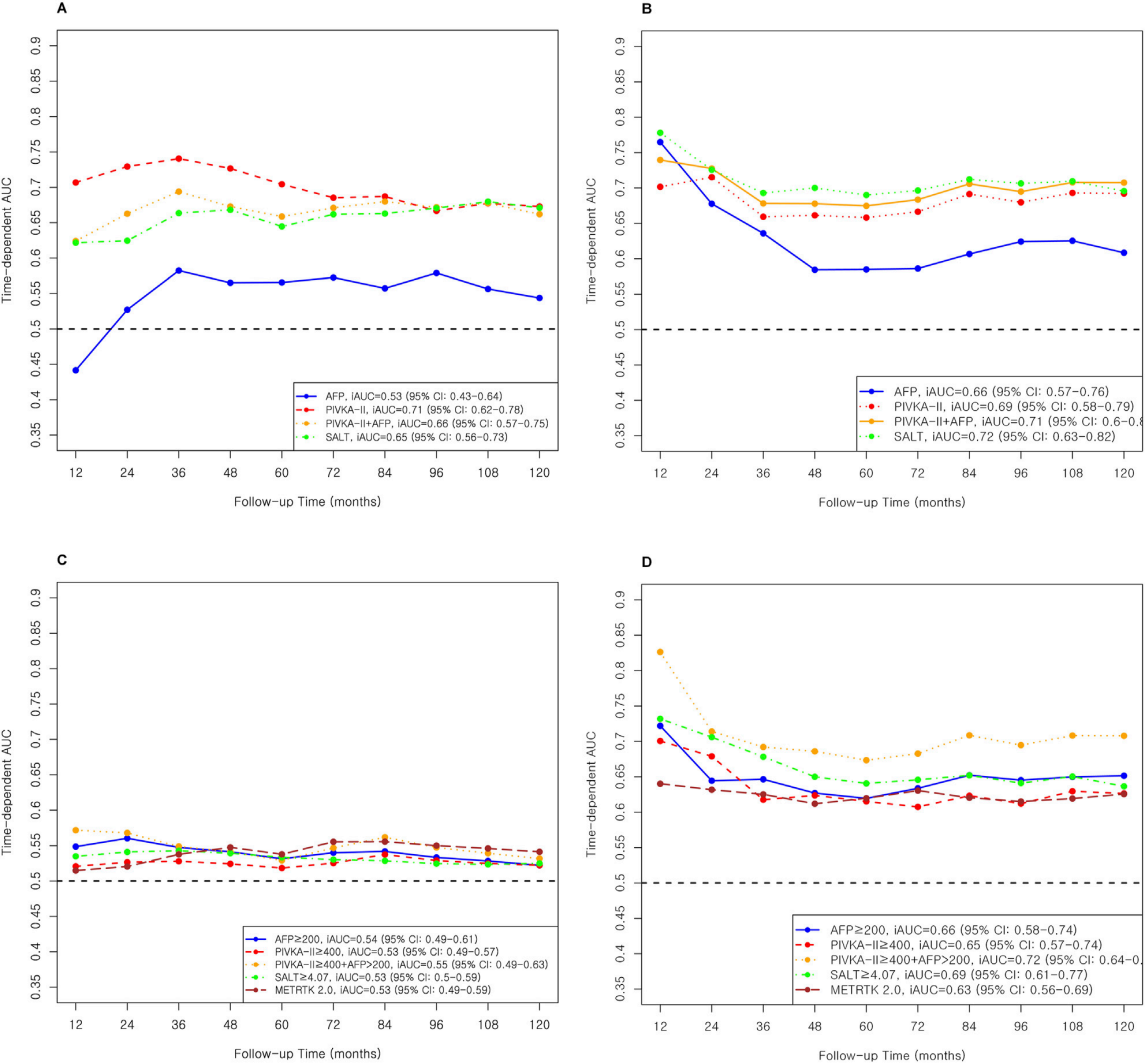
AUC and iAUC for Mortality prediction in Miland and Beyond Milan cohort. **(A)** Continuous variable–based AUC and iAUC in the Milan cohort. **(B)** Continuous variable–based AUC and iAUC in the Beyond Milan cohort. **(C)** Threshold-based AUC and iAUC in the Milan cohort. **(D)** Threshold-based AUC and iAUC in the Beyond Milan cohort.

**FIGURE 5:**
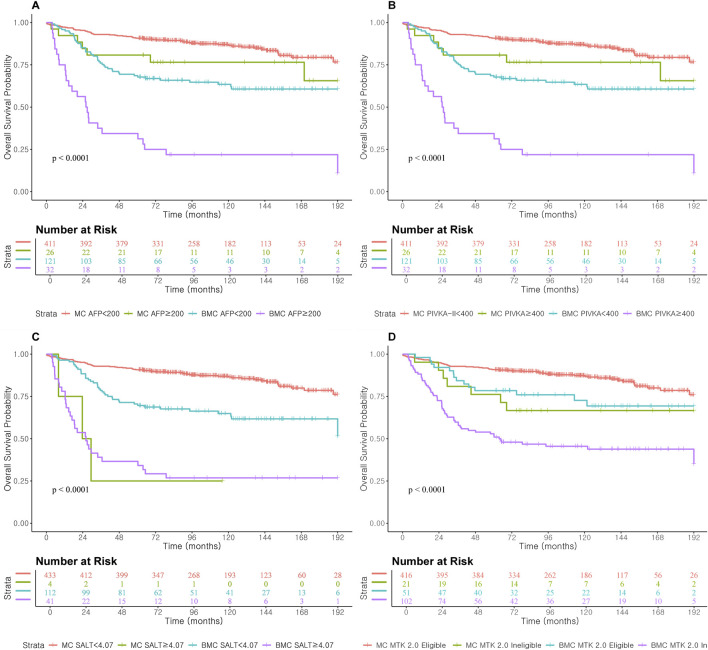
Overall Survival according to prognostic score cut-off and AFP and PIVKA-II cut-off level in Milan and Beyond Milan cohort. **(A)** Overall Survival according to AFP ≥200. **(B)** Overall Survival according to PIVKA-II ≥400. **(C)** Overall Survival according to SALT ≥4.07. **(D)** Overall Survival according to METROTICKET 2.0.

In mortality prediction, PIVKA-II (iAUC 0.71, Table 4; Figure 4) outperformed AFP (0.53) in Milan, with improvement when combined (0.66). SALT ≥4.07 had the highest C-index in Milan (0.86; 95% CI: 0.72–1.00). In Beyond Milan (Table 4; Figure 4), SALT and METROTICKET 2.0 showed C-indices of 0.80 (95% CI: 0.71–0.89) and 0.74 (95% CI: 0.62–0.86), respectively. Overall survival rates for each model are presented in Figure 5.

**TABLE 4:** Integrated AUC and C-index of mortality prediction for both Milan and Beyond Milan cohorts.

Prediction Cox Models	Milan Cohort (N = 437)		Beyond Milan Cohort (N = 153)	
	Mortality		Mortality	
	iAUC (95% CI)	C-index (95% CI)	iAUC (95% CI)	C-index (95% CI)
AFP (continuous)	0.53 (0.44–0.64)	0.6 (0.53–0.68)	0.66 (0.57–0.76)	0.61 (0.55–0.66)
AFP ≥200 (threshold)	0.54 (0.49–0.61)	0.72 (0.54–0.89)	0.66 (0.58–0.74)	0.73 (0.62–0.84)
PIVKA-II (continuous)	0.71 (0.62–0.78)	0.66 (0.59–0.73)	0.69 (0.58–0.78)	0.62 (0.55–0.69)
PIVKA-II ≥400 (threshold)	0.53 (0.49–0.57)	0.79 (0.65–0.93)	0.65 (0.57–0.73)	0.74 (0.63–0.85)
PIVKA-II + AFP (continuous)	0.66 (0.57–0.75)	0.67 (0.61–0.74)	0.71 (0.61–0.8)	0.64 (0.57–0.71)
PIVKA-II ≥400+AFP ≥200 (threshold)	0.55 (0.49–0.62)	0.72 (0.59–0.86)	0.72 (0.64–0.81)	0.73 (0.65–0.81)
SALT (continuous)	0.65 (0.56–0.73)	0.66 (0.59–0.73)	0.72 (0.63–0.82)	0.67 (0.61–0.74)
SALT ≥4.07 (threshold)	0.53 (0.5–0.59)	0.86 (0.72–1)	0.69 (0.61–0.77)	0.8 (0.71–0.89)
METROTICKET 2.0 (threshold)	0.53 (0.49–0.58)	0.74 (0.6–0.88)	0.63 (0.56–0.69)	0.74 (0.62–0.86)

iAUC, integrated area under curve; C-index, concordance index; 95%CI, 95% confidence interval; AFP, alpha-fetoprotein; PIVKA-II, protein induced by vitamin K absence-II; SALT, survival after liver transplantation.

### Post-LT Location Specific HCC Recurrence Prediction

We further analyzed location-specific HCC recurrence by intrahepatic and extrahepatic patterns (Supplementary Figures S1,S2; Supplementary Table S2). In the Milan cohort, complex prognostic models incorporating tumor morphology, vascular invasion, and PET metabolic activity showed improved performance for intrahepatic recurrence. R3-AFP achieved an iAUC of 0.82 (95% CI 0.63–0.94), RETREAT ≥5 a C-index of 0.93 (95% CI 0.79–1.00), and SNAPP ≥5 a C-index of 0.96 (95% CI 0.85–1.00). In the Beyond Milan cohort, R3-AFP yielded an iAUC of 0.68 (95% CI 0.52–0.82), SNAPP ≥5 a C-index of 0.71 (95% CI 0.52–0.90), and MoRAL ≥314.8 a C-index of 0.78 (95% CI 0.61–0.94).

For extrahepatic recurrence in the Milan cohort (Supplementary Figure S2; Supplementary Table S2), AFP and PIVKA-II showed iAUCs of 0.56 (95% CI 0.45–0.67) and 0.66 (95% CI 0.57–0.75), respectively. The combined AFP + PIVKA-II model reached an iAUC of 0.61 (95% CI 0.51–0.70). Composite models outperformed individual markers: R3-AFP reached 0.60 (95% CI 0.49–0.72), and SNAPP ≥5 achieved the highest C-index at 0.81 (95% CI 0.56–1.00). In the Beyond Milan cohort, R3-AFP showed the highest iAUC at 0.83 (95% CI 0.75–0.89), followed by SNAPP at 0.77 (95% CI 0.67–0.96) and AFP + PIVKA-II at 0.73 (95% CI 0.62–0.92). Threshold models R3-AFP ≥3 and SNAPP ≥5 demonstrated C-indices of 0.86 (95% CI 0.76–0.95) and 0.80 (95% CI 0.71–0.90), respectively.

Overall, complex prognostic models provided higher predictive accuracy for both intrahepatic and extrahepatic recurrence than biomarker-only approaches (Supplementary Table S2). Performance was generally higher in the Beyond Milan cohort.

## Discussion

The current study evaluated the predictive performance of AFP, PIVKA-II, and multiple prognostic models for HCC recurrence (RETREAT, MoRAL, SNAPP, and R3-AFP) and mortality (SALT and METROTICKET 2.0) in both MC and BMC over a 10-year period (Table 3). PIVKA-II exhibited consistently strong predictive performance across both cohorts. Among the complex models, R3-AFP, MoRAL, and SALT demonstrated high accuracy based on the iAUC and C-index values. The combined AFP and PIVKA-II model showed modest gains in the MC but performed comparably to the complex scores in the BMC. Continuous Cox models yielded higher iAUC values than threshold-based models by capturing the full biomarker variability, enabling precise risk estimation. Threshold models, such as AFP ≥200 or PIVKA-II ≥400, showed lower iAUCs but maintained moderate-to-high C-indices in the BMC.

### HCC Recurrence Prediction Performance of Cox Models

Single tumor markers, including PIVKA-II and AFP, showed comparable predictive accuracy to selected multivariable models in post-LT recurrence prediction. In the Milan cohort, PIVKA-II achieved higher iAUC than AFP (0.68 vs. 0.58, Table 3; Figures 2A,C), while both performed similarly in the Beyond Milan cohort (0.68 vs. 0.69). AFP showed better early discrimination but declined after 2 years, whereas SNAPP, MoRAL, and R3-AFP remained stable [3, 28, 29, 38–41]. AFP was limited in long-term prediction. Combining AFP and PIVKA-II improved prediction in Beyond Milan (iAUC: 0.72), comparable to SNAPP and MoRAL (0.72–0.73). In threshold models, PIVKA-II ≥400 had the highest C-index in Milan (0.73), whereas AFP ≥200 performed better in Beyond Milan (0.76). SNAPP ≥5 showed the highest C-index in Milan (0.88). While AFP remains widely used, dynamic assessment offers better early prediction but weaker long-term value [3, 28, 29, 38–41]. PIVKA-II shows independent accuracy, particularly for early recurrence [36, 40, 42, 43]. Continuous models offer time-sensitive monitoring advantages over fixed thresholds, and AFP and PIVKA-II integration improves risk stratification post-LT [1, 40].

The R3-AFP model, developed using data from 47 Euro-American centers, showed C-index values of 0.76–0.78 in external validation [17]. In this study, it showed the strongest long-term recurrence prediction among evaluated models (iAUC: 0.64 in Milan and 0.79 in Beyond Milan, Table 3; Figures 2A,B), and strong threshold performance (C-index: 0.76 in Milan and 0.77 in Beyond Milan). Although initially developed in DDLT populations, R3-AFP generalized well to LDLT settings. The incorporation of tumor burden and pathology appears to enhance its predictive power compared to single markers [17]. A recent study validated R3-AFP’s prognostic value in LT recipients with mammalian target of rapamycin inhibitor (mTORi)-based immunosuppression, which could potentially decrease HCC recurrence and improve survival [44].

The SNAPP score showed limited performance as a continuous Cox model in the MC (iAUC: 0.58; C-index: 0.57, Table 3; Figure 2) and BMC. However, SNAPP ≥5 performed better, with C-indices of 0.88 in MC and 0.78 in BMC. For intra-hepatic recurrence, continuous SNAPP had the second highest iAUC (0.73) after R3-AFP (0.82), whereas its threshold model achieved the highest C-index (0.96) in the MC. For extrahepatic recurrence, SNAPP ≥5 maintained C-indices above 0.80 in both cohorts (Supplementary Table S2; Supplementary Figure S2). These results align with the model’s design for LDLT populations in HBV-endemic Asian regions [15, 45]. Prior validation showed a C-index of 0.84 [15]; however, as SNAPP was developed for 5-year recurrence prediction, its fifth-year C-indices were below 80% in both cohorts (Supplementary Tables S1, S2).

The MoRAL score, developed in our center to assess recurrence risk beyond Milan criteria previously, was based on data collected between 2001 and 2013 [14]. Although a C-index above 80% was expected, the BMC showed a C-index of 0.75 (Table 3; Figure 2). The 10-year iAUC of MoRAL was 0.65 for BMC, similar to that of AFP and PIVKA-II. For intra-hepatic recurrence, MoRAL showed lower performance (iAUC 0.64), whereas MoRAL ≥314.8 achieved better discrimination (C-index 0.78). Lower early AUCs may reflect the model’s focus on tumor markers. Studies have validated MoRAL in hepatectomy [46], and deep learning integration has improved accuracy [47]. However, partial data overlap with the original cohort requires further validation [14].

RETREAT, which was primarily validated in DDLT populations, was evaluated in the MC, aligning with its original purpose [16]. The continuous RETREAT model showed an iAUC of 0.60, which was lower than that of PIVKA-II but similar to those of R3-AFP and AFP + PIVKA-II combination. Its threshold version (RETREAT ≥5) performed better, with a C-index of 0.75, ranking third after SNAPP ≥5 and R3-AFP ≥3 (Table 3; Figure 2). For intra-hepatic recurrence, the continuous model showed moderate accuracy (iAUC 0.70, Supplementary Table S2; Supplementary Figure S1), whereas the threshold model demonstrated excellent discrimination (C-index 0.93). RETREAT has been validated in North American cohorts with strong discrimination [48, 49]. UK data confirmed its utility (C-index 0.77) [50], and European data showed a 10-year prediction capability for low-risk HCC recurrence groups [51]. The recent addition of AFP-L3 and PIVKA-II has improved its prognostic performance [52].

### Mortality Prediction Performance of Cox Models

PIVKA-II, as an individual marker and in combination with AFP, showed strong and consistent predictive abilities for post-LT mortality in both MC and BMCs (Table 4; Figure 4). In the MC, PIVKA-II clearly outperformed AFP, highlighting the growing relevance of tumor biology markers in long-term risk assessment [36, 40, 42, 43]. Together, AFP and PIVKA-II, particularly when combined, offer a practical and accessible option for risk stratification, although multivariable models, such as SALT, remained superior for long-term individualized prognostication.

This retrospective study has several limitations. Selection bias and variations in clinical management between the MC and BMC cohorts may have affected model performance. Differences in tumor biology and the predominance of viral hepatitis in this cohort may limit generalizability to Western populations, where non-viral etiologies such as metabolic associated liver diseases are more common. The higher recurrence and mortality rates observed in BMC may have led to an overestimation of risk. Furthermore, our study did not adjust for differences in recurrence treatments such as TACE or chemotherapy, which may have introduced bias. Additionally, immunosuppressive agents such as mammalian target of rapamycin inhibitors (mTORi) and steroids were not standardized and may have varied during model development, potentially impacting predictive performance. From a methodological standpoint, although Uno’s iAUC and Harrell’s C-index offer robust time-dependent and overall performance assessments, these statistical measures do not directly translate to clinical decision-making. The clinical relevance of modest differences in performance metrics remains uncertain. The prognostic models evaluated were developed under varying conditions. SNAPP and MoRAL were designed for LDLT populations, whereas RETREAT and R3-AFP were validated in DDLT settings. MoRAL and SALT were derived from single-center data, which may introduce institutional bias. Furthermore, the lack of an external validation cohort remains a major limitation. Future multicenter studies are necessary to confirm the generalizability and clinical utility of these findings.

However, this study provides long-term validation of prognostic models and tumor biomarkers for post-transplant outcomes in MC and BMCs, evaluating four recurrence and two mortality models over 5 years. In settings where LDLT recipients return to local care with limited diagnostic access, preoperative AFP and PIVKA-II levels could serve as accessible risk assessment markers. When applied as continuous variables, they showed moderate to strong predictive performance, with PIVKA-II outperforming AFP, particularly for long-term mortality. Their combination improved accuracy, matching complex models in high-risk populations. Their threshold-based models demonstrated performance comparable to that of complex models, particularly in BMC. R3-AFP showed the highest consistent predictive performance, whereas SNAPP, MoRAL, and SALT also performed well for BMC. Prognostic models and tumor biological scores generally performed better, particularly in the BMC cohort, where tumors were morphologically larger.

In conclusion, preoperative PIVKA-II, alone or in combination with AFP, may serve as an accessible long-term risk assessment marker for HCC recurrence and mortality following LDLT. However, AFP and PIVKA-II do not fully replace validated multivariable models, which remain the preferred approach in centers with advanced diagnostic capabilities.

## Data Availability

The datasets presented in this article are not publicly available due to institutional IRB policies requiring prior approval for use. Requests to access the data should be directed to the corresponding author.

## Footnotes

1
https://cran.r-project.org/bin/windows/base/old/4.4.1/

## Abbreviations

AFP, alpha-fetoprotein; DDLT, deceased-donor liver transplantation; EMR, electronic medical record; HCC, hepatocellular carcinoma; iAUC, integrated area under the curve; LDLT, living-donor liver transplantation; LT, liver transplantation; MoRAL, Model for Recurrence After Liver Transplantation; PIVKA-II, protein induced by vitamin K absence-II; R3-AFP, Recurrence-Risk Reassessment AFP; RETREAT, Risk Estimation of Tumor Recurrence After Transplant; SALT, Survival After Liver Transplantation; SNAPP, Size and Number, AFP, PIVKA-II, PET.
