# Supplementary material for: Long-Term Prognostic Value of AFP and PIVKA-II in HCC After Living Donor Liver Transplantation: A Single-Center Retrospective Study
Source: Transpl Int. 2025 Jun 27;38:14748. doi: 10.3389/ti.2025.14748 (PMC12245734; doi:10.3389/ti.2025.14748)
Supplement: Supplementary file 1 [file DataSheet1.pdf]

Supplementary Materials

Supplementary Tables

Supplementary Table 1. Pre-LT interventions and post-LT pathological outcomes 2

Supplementary Table 2. Integrated iAUC and overall C-index of HCC recurrence, and location specific recurrence predictions of both Milan and Beyond Milan cohorts 1

Supplementary Table 3. Prognostic Score Characters and criteria assessment outcomes and target populations 2

Supplementary Table 4. Time-dependent AUC, iAUC and C-index of Recurrence Prediction Models of Milan cohort (also shown in Figure 2A, 2C) 3

Supplementary Table 5. Time-dependent AUC, iAUC and C-index of Recurrence Prediction Models of Beyond Milan cohort (also shown in Figure 2B, 2D) 4

Supplementary Table 6. Time-dependent AUC, iAUC and C-index of Mortality Prediction Models of Milan cohort (also shown in Figure 3A, 3C)... 5

Supplementary Table 7. Time-dependent AUC, iAUC and C-index of Mortality Prediction Models of Beyond Milan cohort (also shown in Figure 3B, 3D) 6

Supplementary Table 8. Time-dependent AUC, iAUC and C-index of Intra-Hepatic Recurrence Prediction Models of Milan cohort (also shown in Figure 4A, 4C) 7

Supplementary Table 9. Time-dependent AUC, iAUC and C-index of Intra-Hepatic Recurrence Prediction Models of Beyond Milan cohort (also shown in Figure 4B, 4D).......... 8

Supplementary Table 10. Time-dependent AUC, iAUC and C-index of Extra-Hepatic Recurrence Prediction Models of Milan cohort (also shown in Figure 5A, 5C) 9

Supplementary Table 11. Time-dependent AUC, iAUC and C-index of Extra-Hepatic Recurrence Prediction Models of Beyond Milan cohort (also shown in Figure 5B, 5D)........ 10

Supplementary figures

Supplementary Figure 1. 11

Supplementary Figure 2. 12

Supplementary Table 1. Pre-LT interventions and post-LT pathological outcomes

Variables	Milan Cohort (N = 437)	Beyond Milan Cohort (N = 153)	p-value
**Pre-LT interventions**			
Hepatectomy	50 (11.4%)	13 (8.5%)	0.31
Transarterial chemoembolization	214 (49.0%)	70 (45.8%)	0.49
Radiofrequency ablation	96 (22.0%)	26 (17.0%)	0.19
PEIT	38 (8.7%)	10 (6.5%)	0.40
**Post-LT outcomes**			
**Bleeding**	109 (24.9%)	32 (20.9%)	0.32
**Biliary stricture**	133 (30.4%)	36 (23.5%)	0.10
**Biliary leak**	24 (5.5%)	10 (6.5%)	0.63
**Infection**	82 (18.8%)	32 (20.9%)	0.56
**Rejection**	48 (11.0%)	17 (11.1%)	0.97
**Explant GRWR**	1.2 ± 0.4	1.1 ± 0.3	0.37
**Explant tumor stage**			<0.01
T1a	105 (24.0%)	0 (0.0%)	
T1b	74 (16.9%)	4 (2.6%)	
T2	124 (28.4%)	96 (62.7%)	
T3	0 (0.0%)	25 (16.3%)	
T4	0 (0.0%)	28 (18.3%)	
Total necrosis	134 (30.7%)	0 (0.0%)	
**Explant tumor differentiation**			<0.01
E-S grade I	55 (12.6%)	12 (7.9%)	
E-S grade II	108 (24.7%)	39 (25.5%)	
E-S grade III	119 (27.2%)	83 (54.2%)	
E-S grade IV	21 (4.8%)	19 (12.4%)	
Total necrosis	134 (30.7%)	0 (0.0%)	
**Pre-LT PET-CT**			<0.01
Isometabolic	386 (88.3%)	85 (55.6%)	
Hypermetabolic	51 (11.7%)	68 (44.4%)	
**Explant number of tumors**	1.5 ± 0.8	3.9 ± 2.8	<0.01
**Explant biggest tumor size**	2.2 ± 1.2	5.0 ± 3.5	<0.01
**Explant vascular invasion**			<0.01
None	405 (92.7%)	83 (54.2%)	
Microvascular	32 (7.3%)	45 (29.4%)	
Macrovascular	0 (0.0%)	25 (16.3%)	

LT, liver transplantation;

Supplementary Table 2. Integrated iAUC and overall C-index of location specific HCC recurrence predictions of both Milan and Beyond Milan cohorts

	Milan Cohort (n = 437)				Beyond Milan Cohort (n = 153)			
	Intra-hepatic Recurrence		Extra-hepatic Recurrence		Intra-hepatic Recurrence		Extra-hepatic Recurrence	
**Prediction Cox Models**	**iAUC (95% CI)**	**C-index (95% CI)**	**iAUC (95% CI)**	**C-index (95% CI)**	**iAUC (95% CI)**	**C-index (95% CI)**	**iAUC (95% CI)**	**C-index (95% CI)**
**AFP (continuous)**	0.65 (0.34-0.87)	0.73 (0.56–0.9)	0.56 (0.45-0.67)	0.61 (0.52–0.71)	0.39 (0.22-0.59)	0.41 (0.28–0.55)	0.71 (0.61-.0.8)	0.64 (0.57–0.71)
**AFP≥200 (threshold)**	0.55 (0.46-0.74)	0.71 (0.27–1)	0.51 (0.47-0.56)	0.71 (0.51–0.91)	0.67 (0.55-0.78)	0.79 (0.63–0.95)	0.68 (0.6-0.76)	0.75 (0.64–0.87)
**PIVKA-II (continuous)**	0.72 (0.57-0.88)	0.71 (0.57–0.85)	0.66 (0.57-0.75)	0.62 (0.53–0.72)	0.64 (0.47-0.78)	0.62 (0.47–0.76)	0.7 (0.59-0.79)	0.62 (0.55–0.7)
**PIVKA-II≥400(threshold)**	0.52 (0.51-0.53)	NA	0.5 (0.48-0.55)	0.78 (0.6–0.96)	0.58 (0.47-0.71)	0.68 (0.47–0.89)	0.65 (0.56-0.74)	0.73 (0.6–0.85)
**PIVKA-II+AFP(continuous)**	0.72 (0.57-0.86)	0.76 (0.65–0.86)	0.61 (0.51-0.7)	0.63 (0.54–0.72)	0.58 (0.4-0.76)	0.58 (0.43–0.74)	0.73(0.62-0.92)	0.65 (0.57–0.72)
**PIVKA-II≥400 +AFP≥200 (threshold)**	0.57 (0.48-0.71)	0.77 (0.46–1)	0.5 (0.46-0.57)	0.69 (0.51–0.88)	0.7 (057-0.82)	0.75 (0.61–0.88)	0.74 (0.64-0.92)	0.73 (0.65–0.82)
**SNAPP (continuous)**	0.73 (0.49-0.93)	0.81 (0.64–0.98)	0.48 (0.37-0.6)	0.49 (0.35–0.64)	0.58 (0.4-0.76)	0.57 (0.41–0.74)	0.77 (0.67-0.96)	0.73 (0.65–0.8)
**SNAPP≥5 (threshold)**	0.58 (0.5-0.76)	0.96(0.85–1)	0.51 (0.5-0.53)	0.81 (0.56–1)	0.63 (0.5-0.76)	0.71(0.52–0.9)	0.73 (0.65-0.9)	0.8 (0.71–0.9)
**RETREAT (continuous)**	0.7 (0.42-0.9)	0.76 (0.57–0.95)	0.58 (0.46-0.71)	0.67 (0.54–0.79)				
**RETREAT≥5 (threshold)**	0.57 (0.49-0.73)	0.93 (0.79–1)	0.51 (0.5-0.51)	NA				
**MoRAL (continuous)**					0.68 (0.52-0.82)	0.64 (0.51–0.78)	0.74(0.64-0.92)	0.66 (0.58–0.73)
**MoRAL≥314.8 (threshold)**					0.63 (0.5-0.76)	0.78 (0.61–0.94)	0.64 (0.55-0.73)	0.74(0.62–0.87)
**R3-AFP (continuous)**	0.82 (0.63-0.94)	0.86 (0.72–0.99)	0.6 (0.49-0.72)	0.7 (0.58–0.81)	0.68 (0.52-0.82)	0.63 (0.48–0.78)	0.83 (0.75-0.89)	0.74 (0.67–0.81)
**R3-AFP≥3 (threshold)**	0.64 (0.48-0.85)	0.87 (0.68–1)	0.51 (0.48-0.56)	0.7 (0.45–0.94)	0.59 (0.46-0.7)	0.57 (0.36–0.78)	0.74 (0.68-0.79)	0.86 (0.76–0.95)

iAUC, integrated area under curve; C-index, concordance index; 95%CI, 95% confidence interval; AFP, alpha-fetoprotein; PIVKA-II, protein induced by vitamin K absence-II; SNAPP, Size and Number, AFP, PIVKA-II, PET; RETREAT, Risk Estimation of Tumor Recurrence After Transplant; MoRAL, Model for Recurrence After Liver Transplantation; R3-AFP, Recurrence-Risk Reassessment AFP

Supplementary Table 3. Prognostic score characteristics and compared populations

	Recurrence Prediction		Mortality Prediction Models	
**Prognostic Score Characteristics**	**Milan Cohort**	**Beyond Milan Cohort**	**Milan Cohort**	**Beyond Milan Cohort**
**1.Individual tumor biological markers**	AFP, PIVKA-II	AFP, PIVKA-II	AFP, PIVKA-II	AFP, PIVKA-II
**2.Tumor biological markers combined**	AFP + PIVKA-II	AFP +PIVKA-II<br>MoRAL	AFP + PIVKA-II	AFP +PIVKA-II
**3.Tumor biological markers**<br>**+ tumor morphology**	-		METROTICKET 2.0<br>transplantability	METROTICKET 2.0<br>transplantability
**4.Tumor biological markers**<br>**+ tumor morphology**<br>**+ vascular invasion status**	R3-AFP<br>RETREAT	R3-AFP		
**5.Tumor biological markers**<br>**+ tumor morphology**<br>**+ positron emission tomography**	SNAPP	SNAPP	SALT	SALT

AFP, alpha-fetoprotein; PIVKA-II, protein induced by vitamin K absence-II; MoRAL, Model for Recurrence After Liver Transplantation; R3-AFP, Recurrence-Risk Reassessment AFP; RETREAT, Risk Estimation of Tumor Recurrence After Transplant; SNAPP, Size and Number, AFP, PIVKA-II, PET; SALT, Survival After Liver Transplantation

Supplementary Table 4. Time-dependent AUC, iAUC and C-index of Recurrence Prediction Models of Milan cohort (also shown in Figure 2A, 2C)

	Time-dependent AUC (95% CI)										Integrated AUC (95%CI)
**Prediction Models**	**1 Year**	**2 Year**	**3 Year**	**4 Year**	**5 Year**	**6 Year**	**7 Year**	**8 Year**	**9 Year**	**10 Year**	
AFP (continuous)	0.56 (0.44–0.67)	0.59 (0.49–0.70)	0.61 (0.52–0.71)	0.60 (0.50–0.70)	0.61 (0.52–0.71)	0.61 (0.51–0.70)	0.61 (0.51–0.70)	0.60 (0.49–0.69)	0.59 (0.48–0.69)	0.56 (0.45–0.67)	0.58 (0.47-0.68)
AFP≥200 (threshold)	0.51 (0.47–0.59)	0.52 (0.47–0.58)	0.53 (0.48–0.59)	0.52 (0.48–0.58)	0.54 (0.49–0.59)	0.54 (0.49–0.60)	0.54 (0.49–0.59)	0.54 (0.49–0.59)	0.53 (0.48–0.58)	0.52 (0.48–0.57)	0.52 (0.48-0.57)
PIVKA-II	0.67 (0.59–0.76)	0.68 (0.60–0.77)	0.69 (0.61–0.77)	0.66 (0.57–0.74)	0.67 (0.59–0.76)	0.67 (0.58–0.75)	0.68 (0.60–0.76)	0.67 (0.59–0.74)	0.67 (0.59–0.75)	0.68 (0.60–0.76)	0.68(0.6-0.75)
PIVKA-II≥400	0.48 (0.47–0.49)	0.51 (0.48–0.56)	0.53 (0.48–0.58)	0.52 (0.49–0.57)	0.52 (0.48–0.57)	0.52 (0.48–0.56)	0.52 (0.48–0.56)	0.52 (0.48–0.56)	0.51 (0.48–0.56)	0.51 (0.48–0.55)	0.5 (0.48-0.53)
PIVKA-II+AFP	0.62 (0.53–0.71)	0.66 (0.57–0.74)	0.68 (0.61–0.76)	0.65 (0.56–0.73)	0.66 (0.58–0.74)	0.65 (0.57–0.74)	0.66 (0.57–0.74)	0.64 (0.56–0.72)	0.64 (0.55–0.74)	0.63 (0.53–0.72)	0.64 (0.55-0.72)
PIVKA-II≥400+AFP≥200	0.50 (0.45–0.57)	0.52 (0.47–0.58)	0.53 (0.47–0.60)	0.52 (0.47–0.58)	0.54 (0.48–0.60)	0.54 (0.48–0.60)	0.54 (0.49–0.59)	0.53 (0.48–0.59)	0.53 (0.48–0.59)	0.52 (0.47–0.58)	0.51 (0.46-0.57)
SNAPP	0.50 (0.37–0.63)	0.58 (0.46–0.68)	0.59 (0.49–0.69)	0.55 (0.46–0.65)	0.57 (0.47–0.66)	0.56 (0.46–0.66)	0.58 (0.49–0.67)	0.58 (0.49–0.67)	0.58 (0.49–0.67)	0.58 (0.49–0.67)	0.54 (0.42-0.65)
SNAPP≥5	0.52 (0.49–0.57)	0.53 (0.50–0.58)	0.53 (0.50–0.57)	0.52 (0.50–0.56)	0.52 (0.50–0.56)	0.52 (0.50–0.56)	0.52 (0.50–0.56)	0.52 (0.50–0.56)	0.52 (0.50–0.56)	0.52 (0.50–0.55)	0.52 (0.5-0.56)
RETREAT	0.58 (0.44–0.71)	0.62 (0.50–0.72)	0.63 (0.54–0.73)	0.64 (0.55–0.74)	0.65 (0.54–0.74)	0.64 (0.54–0.74)	0.64 (0.55–0.73)	0.64 (0.55–0.74)	0.64 (0.54–0.73)	0.63 (0.54–0.72)	0.6 (0.48-0.72)
RETREAT≥5	0.52 (0.49–0.57)	0.51 (0.49–0.54)	0.51 (0.49–0.54)	0.51 (0.49–0.54)	0.51 (0.49–0.54)	0.50 (0.49–0.54)	0.50 (0.49–0.53)	0.50 (0.49–0.53)	0.50 (0.48–0.53)	0.50 (0.49–0.53)	0.51 (0.49-0.55)
R3-AFP	0.63 (0.51–0.75)	0.65 (0.55–0.75)	0.66 (0.56–0.74)	0.67 (0.59–0.75)	0.68 (0.58–0.76)	0.67 (0.59–0.75)	0.69 (0.61–0.77)	0.69 (0.61–0.77)	0.68 (0.60–0.77)	0.66 (0.54–0.75)	0.64 (0.54-0.74)
R3-AFP≥3	0.55 (0.48–0.62)	0.53 (0.48–0.59)	0.54 (0.49–0.59)	0.53 (0.49–0.58)	0.55 (0.50–0.60)	0.54 (0.49–0.60)	0.54 (0.50–0.59)	0.54 (0.49–0.59)	0.54 (0.49–0.59)	0.54 (0.49–0.58)	0.54 (0.48-0.6)

	Dynamic C-index (95% CI)									
**Prediction Models**	**1 Year**	**2 Year**	**3 Year**	**4 Year**	**5 Year**	**6 Year**	**7 Year**	**8 Year**	**9 Year**	**10 Year**
AFP	0.47 (0.33–0.62)	0.46 (0.34–0.58)	0.44 (0.33–0.56)	0.48 (0.37–0.59)	0.47 (0.36–0.57)	0.59 (0.5–0.67)	0.62 (0.54–0.7)	0.63 (0.55–0.71)	0.64 (0.55–0.72)	0.64 (0.56–0.72)
AFP≥200	0.75 (0.5–1)	0.66 (0.36–0.95)	0.58 (0.29–0.88)	0.62 (0.36–0.89)	0.55 (0.27–0.82)	0.57 (0.34–0.8)	0.56 (0.34–0.79)	0.61 (0.4–0.83)	0.67 (0.47–0.87)	0.71 (0.53–0.9)
PIVKA-II	0.5 (0.36–0.63)	0.47 (0.34–0.59)	0.46 (0.36–0.56)	0.51 (0.42–0.61)	0.48 (0.39–0.57)	0.59 (0.51–0.67)	0.6 (0.52–0.68)	0.65 (0.57–0.73)	0.65 (0.57–0.73)	0.64 (0.57–0.72)
PIVKA-II≥400	NA (NA–NA)	0.22 (0.21–0.24)	0.29 (0.18–0.4)	0.36 (0.25–0.47)	0.41 (0.31–0.52)	0.7 (0.64–0.76)	0.61 (0.37–0.84)	0.68 (0.46–0.89)	0.73 (0.53–0.92)	0.73 (0.52–0.93)
PIVKA-II+AFP	0.51 (0.38–0.64)	0.45 (0.34–0.57)	0.42 (0.32–0.53)	0.48 (0.38–0.59)	0.46 (0.36–0.56)	0.61 (0.53–0.7)	0.62 (0.54–0.7)	0.66 (0.59–0.74)	0.66 (0.58–0.73)	0.66 (0.58–0.73)
PIVKA-II≥400+AFP≥200	0.73 (0.48–0.99)	0.54 (0.26–0.82)	0.52 (0.28–0.77)	0.57 (0.35–0.79)	0.53 (0.3–0.75)	0.59 (0.4–0.78)	0.54 (0.34–0.74)	0.6 (0.4–0.79)	0.65 (0.47–0.84)	0.68 (0.5–0.86)
SNAPP	0.48 (0.28–0.67)	0.39 (0.23–0.54)	0.42 (0.27–0.56)	0.5 (0.35–0.64)	0.49 (0.35–0.63)	0.57 (0.45–0.69)	0.54 (0.42–0.66)	0.56 (0.44–0.68)	0.57 (0.45–0.69)	0.57 (0.45–0.7)
SNAPP≥5	NA (NA–NA)	0.65 (0.2–1)	0.71 (0.34–1)	0.74 (0.4–1)	0.76 (0.46–1)	0.87 (0.72–1)	0.8 (0.53–1)	0.84 (0.62–1)	0.86 (0.68–1)	0.88 (0.72–1)
RETREAT	0.52 (0.37–0.67)	0.52 (0.39–0.64)	0.51 (0.38–0.64)	0.51 (0.39–0.64)	0.53 (0.4–0.65)	0.66 (0.55–0.78)	0.68 (0.57–0.79)	0.68 (0.57–0.8)	0.69 (0.58–0.8)	0.69 (0.58–0.8)
RETREAT≥5	NA (NA–NA)	NA (NA–NA)	NA (NA–NA)	NA (NA–NA)	NA (NA–NA)	NA (NA–NA)	0.77 (0.29–1)	0.81 (0.39–1)	0.84 (0.47–1)	0.75 (0.3–1)
R3-AFP	0.5 (0.3–0.71)	0.51 (0.34–0.67)	0.54 (0.39–0.69)	0.55 (0.4–0.69)	0.56 (0.42–0.7)	0.7 (0.59–0.81)	0.71 (0.6–0.82)	0.72 (0.62–0.82)	0.74 (0.64–0.84)	0.74 (0.64–0.83)
R3-AFP≥3	0.74 (0.32–1)	0.82 (0.53–1)	0.69 (0.36–1)	0.72 (0.42–1)	0.63 (0.31–0.94)	0.81 (0.66–0.96)	0.74 (0.55–0.93)	0.76 (0.58–0.93)	0.77 (0.6–0.94)	0.76 (0.58–0.93)

AUC, area under curve; C-index, concordance index; 95% CI, 95% confidence interval; AFP, alpha-fetoprotein; PIVKA-II, protein induced by vitamin K absence-II; SNAPP, Size and Number, AFP, PIVKA-II, PET; RETREAT, Risk Estimation of Tumor Recurrence After Transplant; R3-AFP, Recurrence-Risk Reassessment AFP

Supplementary Table 5. Time-dependent AUC, iAUC and C-index of Recurrence Prediction Models of Beyond Milan cohort (also shown in Figure 2B, 2D)

	**Time-dependent AUC (95% CI)**										**Integrated AUC (95%CI)**
**Prediction Models**	**1 Year**	**2 Year**	**3 Year**	**4 Year**	**5 Year**	**6 Year**	**7 Year**	**8 Year**	**9 Year**	**10 Year**	
AFP	0.73 (0.62–0.83)	0.67 (0.57–0.77)	0.64 (0.55–0.74)	0.61 (0.51–0.70)	0.62 (0.51–0.70)	0.60 (0.50–0.69)	0.60 (0.49–0.69)	0.61 (0.50–0.71)	0.59 (0.47–0.68)	0.59 (0.47–0.68)	0.69 (0.61-0.79)
AFP≥200	0.69 (0.61–0.78)	0.68 (0.62–0.75)	0.66 (0.59–0.73)	0.64 (0.58–0.70)	0.65 (0.59–0.72)	0.64 (0.58–0.70)	0.65 (0.60–0.71)	0.65 (0.59–0.71)	0.66 (0.59–0.71)	0.66 (0.60–0.72)	0.68 (0.61-0.75)
PIVKA-II	0.70 (0.59–0.80)	0.66 (0.57–0.74)	0.68 (0.59–0.77)	0.67 (0.57–0.75)	0.69 (0.59–0.77)	0.68 (0.58–0.76)	0.68 (0.59–0.77)	0.68 (0.58–0.76)	0.69 (0.59–0.78)	0.69 (0.59–0.78)	0.68 (0.58-0.78)
PIVKA-II≥400	0.67 (0.58–0.76)	0.59 (0.52–0.67)	0.62 (0.55–0.69)	0.61 (0.55–0.68)	0.62 (0.55–0.69)	0.61 (0.54–0.68)	0.60 (0.53–0.68)	0.60 (0.52–0.67)	0.61 (0.53–0.68)	0.62 (0.54–0.69)	0.63 (0.56-0.78)
PIVKA-II+AFP	0.73 (0.63–0.83)	0.69 (0.61–0.78)	0.71 (0.63–0.79)	0.70 (0.62–0.78)	0.72 (0.63–0.80)	0.71 (0.62–0.79)	0.71 (0.61–0.79)	0.70 (0.60–0.77)	0.71 (0.61–0.81)	0.72 (0.61–0.80)	0.72 (0.62-0.8)
PIVKA-II≥400+AFP≥200	0.76 (0.67–0.84)	0.71 (0.64–0.79)	0.72 (0.64–0.79)	0.70 (0.63–0.77)	0.71 (0.64–0.78)	0.69 (0.61–0.76)	0.70 (0.62–0.77)	0.69 (0.61–0.76)	0.70 (0.62–0.78)	0.72 (0.64–0.78)	0.74 (0.65-0.82)
SNAPP	0.75 (0.63–0.85)	0.70 (0.61–0.79)	0.71 (0.62–0.79)	0.69 (0.60–0.77)	0.70 (0.60–0.78)	0.68 (0.58–0.76)	0.69 (0.60–0.78)	0.68 (0.59–0.77)	0.68 (0.58–0.76)	0.69 (0.59–0.78)	0.72 (0.63-0.82)
SNAPP≥5	0.72 (0.64–0.81)	0.69 (0.61–0.75)	0.70 (0.63–0.77)	0.68 (0.61–0.75)	0.69 (0.62–0.75)	0.68 (0.60–0.74)	0.68 (0.62–0.75)	0.68 (0.61–0.75)	0.68 (0.60–0.74)	0.69 (0.61–0.75)	0.73 (0.65-0.81)
MoRAL	0.74 (0.64–0.84)	0.71 (0.62–0.79)	0.72 (0.64–0.80)	0.71 (0.63–0.80)	0.72 (0.64–0.80)	0.71 (0.62–0.80)	0.72 (0.61–0.80)	0.71 (0.61–0.79)	0.72 (0.61–0.80)	0.73 (0.62–0.82)	0.65 (0.58-0.73)
MoRAL≥314.8	0.68 (0.60–0.76)	0.61 (0.53–0.68)	0.62 (0.56–0.69)	0.62 (0.55–0.68)	0.63 (0.56–0.70)	0.61 (0.55–0.68)	0.60 (0.53–0.67)	0.60 (0.53–0.67)	0.62 (0.54–0.68)	0.61 (0.54–0.68)	0.65 (0.58-0.73)
R3-AFP	0.82 (0.74–0.90)	0.75 (0.67–0.83)	0.78 (0.70–0.85)	0.78 (0.71–0.85)	0.78 (0.70–0.85)	0.76 (0.68–0.83)	0.75 (0.66–0.83)	0.75 (0.66–0.83)	0.76 (0.67–0.83)	0.77 (0.67–0.85)	0.79 (0.71-0.86)
R3-AFP≥3	0.70 (0.62–0.76)	0.69 (0.61–0.76)	0.70 (0.63–0.77)	0.70 (0.63–0.77)	0.70 (0.63–0.77)	0.68 (0.60–0.75)	0.67 (0.59–0.75)	0.67 (0.59–0.76)	0.68 (0.58–0.76)	0.69 (0.58–0.77)	0.7 (0.63-0.76)

	**Dynamic C-index (95%CI)**									
**Prediction Models**	**1 Year**	**2 Year**	**3 Year**	**4 Year**	**5 Year**	**6 Year**	**7 Year**	**8 Year**	**9 Year**	**10 Year**
AFP	0.59 (0.5–0.67)	0.61 (0.53–0.69)	0.63 (0.57–0.7)	0.65 (0.59–0.72)	0.64 (0.58–0.71)	0.65 (0.59–0.7)	0.64 (0.58–0.69)	0.62 (0.57–0.68)	0.63 (0.57–0.68)	0.63 (0.57–0.68)
AFP≥200	0.64 (0.47–0.81)	0.64 (0.51–0.78)	0.71 (0.59–0.83)	0.74 (0.64–0.85)	0.72 (0.61–0.84)	0.77 (0.68–0.87)	0.75 (0.65–0.85)	0.76 (0.67–0.86)	0.77 (0.67–0.86)	0.76 (0.66–0.86)
PIVKA-II	0.6 (0.5–0.7)	0.61 (0.54–0.69)	0.59 (0.52–0.67)	0.6 (0.52–0.68)	0.59 (0.51–0.66)	0.62 (0.55–0.69)	0.62 (0.55–0.68)	0.63 (0.56–0.69)	0.62 (0.55–0.69)	0.62 (0.55–0.69)
PIVKA-II≥400	0.71 (0.55–0.86)	0.78 (0.66–0.9)	0.68 (0.54–0.82)	0.69 (0.56–0.82)	0.67 (0.54–0.81)	0.73 (0.62–0.85)	0.74 (0.63–0.85)	0.75 (0.65–0.86)	0.72 (0.6–0.83)	0.71 (0.6–0.83)
PIVKA-II+AFP	0.62 (0.51–0.72)	0.62 (0.55–0.69)	0.61 (0.53–0.68)	0.61 (0.54–0.69)	0.6 (0.53–0.68)	0.64 (0.57–0.71)	0.64 (0.57–0.71)	0.65 (0.58–0.72)	0.64 (0.58–0.71)	0.64 (0.58–0.71)
PIVKA-II≥400+AFP≥200	0.66 (0.53–0.79)	0.69 (0.59–0.78)	0.69 (0.61–0.77)	0.71 (0.64–0.79)	0.7 (0.61–0.78)	0.75 (0.68–0.83)	0.74 (0.66–0.81)	0.75 (0.68–0.83)	0.74 (0.66–0.82)	0.73 (0.66–0.81)
SNAPP	0.56 (0.43–0.69)	0.63 (0.56–0.7)	0.63 (0.56–0.7)	0.65 (0.58–0.72)	0.64 (0.58–0.71)	0.69 (0.63–0.76)	0.68 (0.61–0.75)	0.69 (0.62–0.76)	0.69 (0.62–0.76)	0.68 (0.61–0.76)
SNAPP≥5	0.64 (0.47–0.81)	0.69 (0.57–0.82)	0.67 (0.55–0.8)	0.72 (0.61–0.83)	0.71 (0.6–0.82)	0.77 (0.67–0.86)	0.76 (0.66–0.85)	0.77 (0.68–0.86)	0.78 (0.69–0.87)	0.78 (0.69–0.87)
MoRAL	0.62 (0.52–0.72)	0.63 (0.55–0.7)	0.61 (0.54–0.69)	0.62 (0.55–0.7)	0.61 (0.54–0.69)	0.65 (0.58–0.72)	0.65 (0.58–0.71)	0.66 (0.59–0.72)	0.65 (0.59–0.72)	0.65 (0.59–0.72)
MoRAL≥314.8	0.69 (0.53–0.85)	0.76 (0.64–0.89)	0.7 (0.57–0.84)	0.7 (0.57–0.84)	0.69 (0.55–0.82)	0.74 (0.63–0.86)	0.77 (0.66–0.87)	0.78 (0.68–0.88)	0.74 (0.63–0.85)	0.75 (0.65–0.86)
R3-AFP	0.5 (0.4–0.6)	0.64 (0.58–0.7)	0.62 (0.55–0.69)	0.63 (0.56–0.7)	0.64 (0.57–0.71)	0.71 (0.64–0.77)	0.71 (0.64–0.77)	0.72 (0.65–0.78)	0.71 (0.65–0.77)	0.71 (0.64–0.77)
R3-AFP≥3	0.65 (0.52–0.79)	0.65 (0.51–0.79)	0.66 (0.53–0.8)	0.69 (0.56–0.82)	0.7 (0.57–0.82)	0.78 (0.68–0.88)	0.79 (0.69–0.88)	0.78 (0.69–0.88)	0.78 (0.68–0.87)	0.77 (0.67–0.87)

AUC, area under curve; C-index, concordance index; 95% CI, 95% confidence interval; AFP, alpha-fetoprotein; PIVKA-II, protein induced by vitamin K absence-II; SNAPP, Size and Number, AFP, PIVKA-II, PET; R3-AFP, MoRAL, Model for Recurrence After Liver Transplantation; R3-AFP, Recurrence-Risk Reassessment AFP

Supplementary Table 6. Time-dependent AUC, iAUC and C-index of Mortality Prediction Models of Milan cohort (also shown in Figure 3A, 3C)

	Time-dependent AUC (95% CI)										Integrated AUC
**Prediction Models**	**1 Year**	**2 Year**	**3 Year**	**4 Year**	**5 Year**	**6 Year**	**7 Year**	**8 Year**	**9 Year**	**10 Year**	**(95%CI)**
AFP	0.44 (0.28–0.62)	0.53 (0.40–0.66)	0.58 (0.47–0.68)	0.57 (0.47–0.67)	0.57 (0.47–0.65)	0.57 (0.48–0.67)	0.56 (0.46–0.66)	0.58 (0.49–0.67)	0.56 (0.46–0.65)	0.54 (0.45–0.64)	0.53 (0.44-0.64
AFP≥200	0.55 (0.47–0.67)	0.56 (0.49–0.65)	0.55 (0.49–0.61)	0.54 (0.49–0.60)	0.53 (0.49–0.58)	0.54 (0.50–0.59)	0.54 (0.50–0.59)	0.53 (0.49–0.58)	0.53 (0.49–0.57)	0.52 (0.48–0.56)	0.54 (0.49-0.61)
PIVKA-II	0.71 (0.55–0.84)	0.73 (0.63–0.81)	0.74 (0.66–0.81)	0.73 (0.65–0.80)	0.70 (0.63–0.78)	0.69 (0.61–0.76)	0.69 (0.61–0.76)	0.67 (0.58–0.74)	0.68 (0.58–0.76)	0.67 (0.56–0.75)	0.71 (0.62-0.78)
PIVKA-II≥400	0.52 (0.48–0.62)	0.53 (0.48–0.60)	0.53 (0.48–0.58)	0.52 (0.49–0.58)	0.52 (0.48–0.56)	0.53 (0.49–0.57)	0.54 (0.50–0.59)	0.53 (0.49–0.57)	0.52 (0.49–0.57)	0.52 (0.49–0.56)	0.53 (0.49-0.57)
PIVKA-II+AFP	0.63 (0.46–0.79)	0.67 (0.56–0.77)	0.70 (0.61–0.78)	0.68 (0.59–0.75)	0.66 (0.57–0.74)	0.67 (0.60–0.75)	0.68 (0.60–0.76)	0.67 (0.60–0.76)	0.68 (0.60–0.75)	0.66 (0.57–0.75)	0.66 (0.57-0.75)
PIVKA-II≥400+AFP>200	0.57 (0.46–0.70)	0.57 (0.48–0.66)	0.55 (0.48–0.62)	0.54 (0.48–0.61)	0.53 (0.48–0.58)	0.55 (0.50–0.61)	0.56 (0.51–0.62)	0.55 (0.50–0.60)	0.54 (0.49–0.59)	0.53 (0.48–0.58)	0.55 (0.49-0.62)
SALT	0.62 (0.48–0.76)	0.62 (0.52–0.73)	0.66 (0.57–0.75)	0.67 (0.58–0.75)	0.64 (0.55–0.73)	0.66 (0.57–0.75)	0.66 (0.58–0.75)	0.67 (0.60–0.75)	0.68 (0.61–0.76)	0.67 (0.58–0.75)	0.65 (0.56-0.73)
SALT≥4.07	0.53 (0.49–0.63)	0.54 (0.50–0.61)	0.54 (0.50–0.60)	0.54 (0.50–0.59)	0.53 (0.50–0.57)	0.53 (0.50–0.57)	0.53 (0.50–0.56)	0.52 (0.50–0.55)	0.52 (0.50–0.55)	0.52 (0.50–0.55)	0.53 (0.5-0.59)
METRTK 2.0	0.51 (0.47–0.60)	0.52 (0.47–0.59)	0.54 (0.49–0.60)	0.55 (0.49–0.61)	0.54 (0.49–0.59)	0.56 (0.51–0.61)	0.56 (0.51–0.61)	0.55 (0.51–0.60)	0.55 (0.50–0.59)	0.54 (0.50–0.58)	0.53 (0.49-0.58)

	Dynamic C-index (95%CI)									
**Prediction Models**	**1 Year**	**2 Year**	**3 Year**	**4 Year**	**5 Year**	**6 Year**	**7 Year**	**8 Year**	**9 Year**	**10 Year**
AFP	–	0.34 (0.26–0.43)	0.36 (0.27–0.45)	0.41 (0.31–0.52)	0.44 (0.34–0.54)	0.57 (0.49–0.65)	0.6 (0.52–0.67)	0.6 (0.52–0.68)	0.6 (0.53–0.68)	0.6 (0.53–0.68)
AFP≥200	–	0.33 (0.04–0.62)	0.5 (0.3–0.71)	0.55 (0.36–0.74)	0.62 (0.46–0.78)	0.6 (0.38–0.82)	0.55 (0.34–0.77)	0.61 (0.41–0.82)	0.67 (0.48–0.86)	0.72 (0.54–0.89)
PIVKA-II	–	0.51 (0.36–0.66)	0.52 (0.4–0.63)	0.56 (0.45–0.67)	0.58 (0.47–0.69)	0.65 (0.58–0.73)	0.64 (0.57–0.72)	0.68 (0.61–0.75)	0.68 (0.61–0.75)	0.66 (0.59–0.73)
PIVKA-II≥400	–	0.4 (0–0.94)	0.48 (0.18–0.79)	0.53 (0.25–0.81)	0.6 (0.36–0.84)	0.73 (0.57–0.9)	0.66 (0.46–0.86)	0.74 (0.58–0.9)	0.79 (0.65–0.93)	0.79 (0.65–0.93)
PIVKA-II+AFP	–	0.48 (0.33–0.63)	0.47 (0.37–0.57)	0.52 (0.41–0.63)	0.53 (0.43–0.64)	0.64 (0.56–0.72)	0.65 (0.57–0.72)	0.68 (0.61–0.75)	0.68 (0.61–0.75)	0.67 (0.61–0.74)
PIVKA-II≥400+AFP>200	–	0.43 (0.13–0.74)	0.56 (0.35–0.77)	0.6 (0.41–0.79)	0.66 (0.49–0.83)	0.63 (0.46–0.81)	0.57 (0.39–0.74)	0.64 (0.48–0.8)	0.7 (0.55–0.84)	0.72 (0.59–0.86)
SALT	–	0.45 (0.32–0.58)	0.41 (0.31–0.52)	0.43 (0.33–0.52)	0.48 (0.38–0.58)	0.63 (0.56–0.7)	0.65 (0.58–0.72)	0.67 (0.6–0.74)	0.66 (0.59–0.74)	0.66 (0.59–0.73)
SALT≥4.07	–	0.29 (0–0.66)	0.39 (0.12–0.65)	0.44 (0.2–0.69)	0.52 (0.31–0.74)	0.78 (0.68–0.88)	0.85 (0.78–0.92)	0.88 (0.83–0.94)	0.91 (0.86–0.95)	0.86 (0.72–1)
METRTK 2.0	–	0.29 (0–0.66)	0.34 (0.13–0.56)	0.31 (0.11–0.52)	0.42 (0.24–0.6)	0.64 (0.5–0.79)	0.63 (0.46–0.79)	0.63 (0.46–0.81)	0.7 (0.54–0.85)	0.74 (0.6–0.88)

AUC, area under curve; C-index, concordance index; 95% CI, 95% confidence interval; AFP, alpha-fetoprotein; PIVKA-II, protein induced by vitamin K absence-II; SALT, Survival After Liver Transplantation; METRK 2.0, METROTICKET 2.0 transplantability

Supplementary Table 7. Time-dependent AUC, iAUC and C-index of Mortality Prediction Models of Beyond Milan cohort (also shown in Figure 3B, 3D)

	Time-dependent AUC (95% CI)										Integrated AUC (95%CI)
**Prediction Models**	**1 Year**	**2 Year**	**3 Year**	**4 Year**	**5 Year**	**6 Year**	**7 Year**	**8 Year**	**9 Year**	**10 Year**	
AFP	0.76 (0.63–0.90)	0.68 (0.56–0.79)	0.64 (0.54–0.73)	0.58 (0.48–0.68)	0.58 (0.49–0.69)	0.59 (0.48–0.69)	0.61 (0.51–0.71)	0.62 (0.52–0.72)	0.63 (0.52–0.73)	0.61 (0.50–0.71)	0.66 (0.57-0.76)
AFP≥200	0.72 (0.57–0.87)	0.64 (0.56–0.73)	0.65 (0.57–0.72)	0.63 (0.56–0.70)	0.62 (0.55–0.69)	0.63 (0.56–0.70)	0.65 (0.59–0.72)	0.65 (0.58–0.72)	0.65 (0.58–0.72)	0.65 (0.58–0.71)	0.66 (0.58-0.74)
PIVKA-II	0.70 (0.51–0.87)	0.72 (0.59–0.83)	0.66 (0.56–0.76)	0.66 (0.57–0.75)	0.66 (0.56–0.75)	0.67 (0.58–0.75)	0.69 (0.60–0.78)	0.68 (0.58–0.76)	0.69 (0.59–0.78)	0.69 (0.58–0.78)	0.69 (0.58-0.78)
PIVKA-II≥400	0.70 (0.56–0.84)	0.68 (0.59–0.77)	0.62 (0.54–0.70)	0.62 (0.55–0.69)	0.62 (0.54–0.69)	0.61 (0.53–0.69)	0.62 (0.55–0.70)	0.61 (0.54–0.69)	0.63 (0.56–0.70)	0.63 (0.55–0.70)	0.65 (0.57-0.73)
PIVKA-II+AFP	0.74 (0.57–0.88)	0.73 (0.61–0.83)	0.68 (0.58–0.77)	0.68 (0.59–0.77)	0.67 (0.58–0.77)	0.68 (0.59–0.77)	0.71 (0.63–0.80)	0.69 (0.60–0.78)	0.71 (0.62–0.80)	0.71 (0.61–0.80)	0.71 (0.61-0.8)
PIVKA-II≥400+AFP>200	0.83 (0.70–0.91)	0.71 (0.62–0.81)	0.69 (0.60–0.78)	0.69 (0.61–0.77)	0.67 (0.59–0.75)	0.68 (0.60–0.76)	0.71 (0.63–0.78)	0.69 (0.62–0.77)	0.71 (0.63–0.78)	0.71 (0.62–0.78)	0.72 (0.64-0.81)
SALT	0.78 (0.63–0.91)	0.73 (0.60–0.84)	0.69 (0.59–0.79)	0.70 (0.61–0.79)	0.69 (0.58–0.78)	0.70 (0.61–0.79)	0.71 (0.62–0.80)	0.71 (0.62–0.79)	0.71 (0.61–0.79)	0.70 (0.58–0.79)	0.72 (0.63-0.82)
SALT≥4.07	0.73 (0.59–0.86)	0.71 (0.61–0.80)	0.68 (0.60–0.75)	0.65 (0.57–0.72)	0.64 (0.57–0.72)	0.65 (0.57–0.72)	0.65 (0.58–0.73)	0.64 (0.56–0.72)	0.65 (0.58–0.73)	0.64 (0.55–0.71)	0.69 (0.61-0.77)
METRTK 2.0	0.64 (0.54–0.71)	0.63 (0.55–0.70)	0.63 (0.56–0.69)	0.61 (0.54–0.68)	0.62 (0.55–0.69)	0.63 (0.55–0.70)	0.62 (0.54–0.69)	0.62 (0.53–0.69)	0.62 (0.53–0.69)	0.63 (0.53–0.71)	0.63 (0.56-0.69)

	Dynamic C-index (95%CI)									
**Prediction Models**	**1 Year**	**2 Year**	**3 Year**	**4 Year**	**5 Year**	**6 Year**	**7 Year**	**8 Year**	**9 Year**	**10 Year**
AFP	–	0.64 (0.57–0.71)	0.65 (0.57–0.73)	0.67 (0.59–0.74)	0.66 (0.59–0.73)	0.64 (0.58–0.69)	0.61 (0.56–0.67)	0.6 (0.54–0.66)	0.6 (0.54–0.66)	0.61 (0.55–0.66)
AFP≥200	–	0.76 (0.61–0.91)	0.67 (0.52–0.82)	0.71 (0.58–0.84)	0.73 (0.6–0.85)	0.74 (0.63–0.86)	0.71 (0.59–0.82)	0.73 (0.62–0.84)	0.73 (0.62–0.84)	0.73 (0.62–0.84)
PIVKA-II	–	0.55 (0.44–0.67)	0.6 (0.52–0.68)	0.59 (0.51–0.67)	0.59 (0.52–0.67)	0.62 (0.55–0.7)	0.61 (0.54–0.69)	0.63 (0.55–0.7)	0.62 (0.55–0.69)	0.62 (0.55–0.69)
PIVKA-II≥400	–	0.67 (0.47–0.86)	0.76 (0.63–0.89)	0.71 (0.57–0.84)	0.72 (0.59–0.85)	0.77 (0.67–0.88)	0.76 (0.65–0.87)	0.78 (0.68–0.88)	0.74 (0.63–0.85)	0.74 (0.63–0.85)
PIVKA-II+AFP	–	0.56 (0.46–0.67)	0.61 (0.53–0.69)	0.6 (0.52–0.68)	0.61 (0.53–0.68)	0.64 (0.57–0.71)	0.63 (0.56–0.7)	0.64 (0.58–0.71)	0.64 (0.57–0.71)	0.64 (0.57–0.71)
PIVKA-II≥400+AFP>200	–	0.72 (0.58–0.85)	0.69 (0.6–0.78)	0.69 (0.61–0.77)	0.71 (0.63–0.79)	0.75 (0.67–0.82)	0.72 (0.63–0.8)	0.74 (0.66–0.82)	0.73 (0.64–0.81)	0.73 (0.65–0.81)
SALT	–	0.6 (0.54–0.66)	0.63 (0.57–0.69)	0.62 (0.55–0.69)	0.63 (0.56–0.7)	0.67 (0.6–0.74)	0.66 (0.6–0.73)	0.67 (0.6–0.74)	0.67 (0.6–0.74)	0.67 (0.61–0.74)
SALT≥4.07	–	0.72 (0.54–0.9)	0.72 (0.58–0.85)	0.76 (0.64–0.88)	0.77 (0.66–0.89)	0.79 (0.7–0.89)	0.78 (0.68–0.88)	0.8 (0.71–0.89)	0.78 (0.69–0.88)	0.8 (0.71–0.89)
METRTK 2.0	–	0.71 (0.5–0.91)	0.72 (0.53–0.9)	0.74 (0.58–0.89)	0.7 (0.55–0.86)	0.73 (0.6–0.86)	0.75 (0.63–0.87)	0.76 (0.65–0.88)	0.76 (0.64–0.88)	0.74 (0.62–0.86)

AUC, area under curve; C-index, concordance index; 95% CI, 95% confidence interval; AFP, alpha-fetoprotein; PIVKA-II, protein induced by vitamin K absence-II; SALT, Survival After Liver Transplantation; METRK 2.0, METROTICET 2.0 transplantability

Supplementary Table 8. Time-dependent AUC, iAUC and C-index of Intra-Hepatic Recurrence Prediction Models of Milan cohort (also shown in Figure 4A, 4C)

	Time-dependent AUC (95% CI)										Integrated AUC
**Model**	**1 Year**	**2 Year**	**3 Year**	**4 Year**	**5 Year**	**6 Year**	**7 Year**	**8 Year**	**9 Year**	**10 Year**	**(95%CI)**
AFP	0.58 (0.00–0.96)	0.69 (0.48–0.84)	0.70 (0.48–0.85)	0.70 (0.49–0.85)	0.70 (0.49–0.85)	0.69 (0.48–0.86)	0.72 (0.51–0.86)	0.71 (0.50–0.86)	0.70 (0.53–0.86)	0.69 (0.49–0.84)	0.65 (0.34-0.87)
AFP≥200	0.59 (0.46–0.97)	0.52 (0.46–0.65)	0.53 (0.46–0.65)	0.53 (0.46–0.64)	0.53 (0.47–0.65)	0.53 (0.47–0.64)	0.53 (0.47–0.63)	0.52 (0.47–0.62)	0.52 (0.46–0.63)	0.52 (0.46–0.63)	0.55 (0.46-0.74)
PIVKA-II	0.72 (0.49–0.95)	0.73 (0.56–0.88)	0.73 (0.56–0.87)	0.73 (0.56–0.87)	0.74 (0.57–0.88)	0.74 (0.57–0.87)	0.72 (0.59–0.85)	0.71 (0.57–0.84)	0.71 (0.57–0.85)	0.72 (0.59–0.85)	0.72 (0.57-0.88)
PIVKA-II≥400	0.52 (0.51–0.53)	0.52 (0.51–0.53)	0.52 (0.51–0.53)	0.52 (0.51–0.52)	0.52 (0.51–0.53)	0.52 (0.51–0.53)	0.52 (0.51–0.52)	0.52 (0.51–0.53)	0.52 (0.51–0.53)	0.52 (0.51–0.54)	0.52 (0.51-0.53)
PIVKA-II+AFP	0.68 (0.42–0.96)	0.75 (0.64–0.85)	0.75 (0.65–0.86)	0.75 (0.64–0.85)	0.75 (0.65–0.86)	0.75 (0.62–0.84)	0.76 (0.66–0.86)	0.75 (0.62–0.84)	0.75 (0.65–0.85)	0.74 (0.63–0.83)	0.72 (0.57-0.86)
PIVKA-II≥400+AFP≥200	0.61 (0.48–0.97)	0.54 (0.48–0.65)	0.54 (0.48–0.65)	0.54 (0.48–0.65)	0.54 (0.48–0.66)	0.55 (0.48–0.66)	0.54 (0.48–0.64)	0.54 (0.49–0.65)	0.54 (0.49–0.64)	0.54 (0.48–0.64)	0.57 (0.48-0.71)
SNAPP	0.68 (0.21–0.99)	0.76 (0.57–0.92)	0.77 (0.57–0.91)	0.76 (0.58–0.92)	0.76 (0.57–0.92)	0.76 (0.58–0.92)	0.75 (0.58–0.88)	0.75 (0.58–0.90)	0.75 (0.59–0.89)	0.74 (0.59–0.87)	0.73 (0.49-0.93)
SNAPP≥5	0.62 (0.49–0.87)	0.55 (0.50–0.67)	0.55 (0.50–0.65)	0.55 (0.50–0.66)	0.55 (0.50–0.69)	0.55 (0.50–0.66)	0.55 (0.50–0.65)	0.55 (0.50–0.64)	0.55 (0.50–0.65)	0.55 (0.50–0.65)	0.58 (0.5-0.76)
RETREAT	0.69 (0.07–0.99)	0.70 (0.50–0.86)	0.71 (0.51–0.86)	0.71 (0.50–0.86)	0.71 (0.51–0.87)	0.70 (0.51–0.87)	0.71 (0.53–0.85)	0.71 (0.51–0.84)	0.71 (0.52–0.84)	0.71 (0.54–0.85)	0.7 (0.42-0.9)
RETREAT≥5	0.61 (0.49–0.99)	0.55 (0.49–0.66)	0.55 (0.49–0.66)	0.55 (0.49–0.68)	0.55 (0.49–0.66)	0.55 (0.49–0.65)	0.54 (0.49–0.64)	0.54 (0.49–0.65)	0.54 (0.48–0.64)	0.54 (0.48–0.63)	0.57 (0.49-0.73)
R3-AFP	0.89 (0.65–1.00)	0.77 (0.59–0.91)	0.77 (0.58–0.93)	0.77 (0.58–0.91)	0.78 (0.60–0.91)	0.77 (0.60–0.92)	0.79 (0.64–0.91)	0.79 (0.63–0.92)	0.79 (0.63–0.91)	0.79 (0.64–0.91)	0.82 (0.63-0.94)
R3-AFP≥3	0.73 (0.48–0.98)	0.59 (0.48–0.73)	0.59 (0.48–0.74)	0.59 (0.48–0.74)	0.59 (0.48–0.74)	0.59 (0.48–0.74)	0.58 (0.48–0.72)	0.58 (0.48–0.71)	0.58 (0.48–0.72)	0.58 (0.48–0.71)	0.64 (0.48-0.85)

	Dynamic C-index (95%CI)									
**Prediction Models**	**1 Year**	**2 Year**	**3 Year**	**4 Year**	**5 Year**	**6 Year**	**7 Year**	**8 Year**	**9 Year**	**10 Year**
AFP	0.76 (0.54–0.99)	0.61 (0.36–0.87)	0.53 (0.28–0.78)	0.56 (0.34–0.79)	0.55 (0.32–0.77)	0.68 (0.5–0.86)	0.7 (0.53–0.87)	0.72 (0.55–0.89)	0.72 (0.56–0.89)	0.73 (0.56–0.9)
AFP≥200	NA (NA–NA)	0.91 (0.72–1)	0.78 (0.36–1)	0.79 (0.4–1)	0.7 (0.23–1)	0.62 (0.11–1)	0.59 (0.07–1)	0.62 (0.12–1)	0.67 (0.21–1)	0.71 (0.27–1)
PIVKA-II	0.76 (0.34–1)	0.59 (0.3–0.89)	0.56 (0.31–0.8)	0.61 (0.4–0.82)	0.57 (0.36–0.79)	0.68 (0.5–0.86)	0.68 (0.51–0.85)	0.73 (0.58–0.87)	0.72 (0.58–0.86)	0.71 (0.57–0.85)
PIVKA-II≥400	NA (NA–NA)	NA (NA–NA)	NA (NA–NA)	NA (NA–NA)	NA (NA–NA)	NA (NA–NA)	NA (NA–NA)	NA (NA–NA)	NA (NA–NA)	NA (NA–NA)
PIVKA-II+AFP	0.76 (0.42–1)	0.59 (0.28–0.91)	0.53 (0.26–0.79)	0.58 (0.36–0.81)	0.56 (0.34–0.78)	0.72 (0.58–0.86)	0.71 (0.59–0.83)	0.75 (0.65–0.86)	0.75 (0.64–0.86)	0.76 (0.65–0.86)
PIVKA-II≥400+AFP≥200	NA (NA–NA)	NA (NA–NA)	NA (NA–NA)	NA (NA–NA)	0.89 (0.66–1)	0.72 (0.33–1)	0.69 (0.33–1)	0.7 (0.34–1)	0.74 (0.4–1)	0.77 (0.46–1)
SNAPP	0.84 (0.53–1)	0.67 (0.31–1)	0.69 (0.4–0.99)	0.75 (0.51–0.99)	0.74 (0.51–0.98)	0.8 (0.63–0.98)	0.78 (0.6–0.96)	0.8 (0.62–0.97)	0.8 (0.64–0.97)	0.81 (0.64–0.98)
SNAPP≥5	NA (NA–NA)	0.92 (0.73–1)	0.93 (0.77–1)	0.94 (0.79–1)	0.94 (0.81–1)	0.97 (0.89–1)	0.92 (0.75–1)	0.94 (0.8–1)	0.95 (0.83–1)	0.96 (0.85–1)
RETREAT	0.81 (0.43–1)	0.72 (0.37–1)	0.67 (0.35–1)	0.66 (0.35–0.98)	0.66 (0.36–0.96)	0.75 (0.53–0.97)	0.75 (0.54–0.95)	0.76 (0.56–0.96)	0.76 (0.56–0.96)	0.76 (0.57–0.95)
RETREAT≥5	NA (NA–NA)	NA (NA–NA)	NA (NA–NA)	NA (NA–NA)	NA (NA–NA)	NA (NA–NA)	0.94 (0.8–1)	0.95 (0.84–1)	0.96 (0.87–1)	0.93 (0.79–1)
R3-AFP	0.88 (0.72–1)	0.84 (0.68–0.99)	0.81 (0.65–0.98)	0.81 (0.64–0.97)	0.8 (0.63–0.96)	0.85 (0.71–0.99)	0.85 (0.71–0.99)	0.85 (0.71–0.99)	0.86 (0.73–1)	0.86 (0.72–0.99)
R3-AFP≥3	NA (NA–NA)	NA (NA–NA)	0.88 (0.66–1)	0.89 (0.69–1)	0.83 (0.56–1)	0.91 (0.77–1)	0.86 (0.66–1)	0.87 (0.68–1)	0.88 (0.7–1)	0.87 (0.68–1)

AUC, area under curve; C-index, concordance index; 95% CI, 95% confidence interval; AFP, alpha-fetoprotein; PIVKA-II, protein induced by vitamin K absence-II; SNAPP, Size and Number, AFP, PIVKA-II, PET; RETREAT, Risk Estimation of Tumor Recurrence After Transplant; R3-AFP, Recurrence-Risk Reassessment AFP

Supplementary Table 9. Time-dependent AUC, iAUC and C-index of Intra-Hepatic Recurrence Prediction Models of Beyond Milan cohort (also shown in Figure 4B, 4D)

	Time-dependent AUC (95% CI)										Integrated AUC
**Prediction Models**	**1 Year**	**2 Year**	**3 Year**	**4 Year**	**5 Year**	**6 Year**	**7 Year**	**8 Year**	**9 Year**	**10 Year**	**(95%CI)**
AFP	0.35 (0.15–0.56)	0.40 (0.24–0.60)	0.48 (0.31–0.68)	0.47 (0.31–0.66)	0.47 (0.32–0.66)	0.48 (0.31–0.66)	0.47 (0.32–0.66)	0.46 (0.29–0.65)	0.47 (0.31–0.66)	0.47 (0.30–0.67)	0.39 (0.22-0.59)
AFP≥200	0.69 (0.54–0.84)	0.68 (0.56–0.79)	0.64 (0.53–0.73)	0.63 (0.52–0.72)	0.63 (0.52–0.72)	0.63 (0.51–0.72)	0.64 (0.54–0.72)	0.64 (0.53–0.73)	0.64 (0.54–0.74)	0.65 (0.55–0.74)	0.67 (0.55-0.78)
PIVKA-II	0.64 (0.43–0.83)	0.62 (0.46–0.75)	0.68 (0.54–0.81)	0.67 (0.54–0.79)	0.68 (0.56–0.80)	0.67 (0.54–0.80)	0.69 (0.55–0.81)	0.68 (0.54–0.80)	0.69 (0.55–0.82)	0.70 (0.55–0.82)	0.64 (0.47-0.78)
PIVKA-II≥400	0.62 (0.47–0.78)	0.53 (0.42–0.63)	0.59 (0.48–0.71)	0.58 (0.47–0.68)	0.58 (0.48–0.69)	0.57 (0.46–0.67)	0.57 (0.46–0.68)	0.57 (0.46–0.68)	0.58 (0.48–0.69)	0.59 (0.47–0.71)	0.58 (0.47-0.71)
PIVKA-II+AFP	0.59 (0.36–0.82)	0.55 (0.38–0.71)	0.61 (0.47–0.77)	0.62 (0.47–0.76)	0.62 (0.47–0.78)	0.62 (0.46–0.77)	0.63 (0.48–0.77)	0.62 (0.47–0.76)	0.64 (0.48–0.79)	0.63 (0.46–0.77)	0.58 (0.4-0.76)
PIVKA-II≥400+AFP≥200	0.73 (0.55–0.88)	0.68 (0.53–0.83)	0.69 (0.56–0.81)	0.67 (0.54–0.78)	0.68 (0.55–0.80)	0.67 (0.52–0.79)	0.68 (0.55–0.80)	0.68 (0.53–0.79)	0.69 (0.55–0.81)	0.71 (0.56–0.82)	0.7 (057-0.82)
SNAPP	0.60 (0.36–0.84)	0.55 (0.37–0.69)	0.61 (0.46–0.75)	0.58 (0.42–0.73)	0.59 (0.44–0.72)	0.56 (0.39–0.72)	0.58 (0.42–0.73)	0.57 (0.42–0.73)	0.57 (0.41–0.72)	0.58 (0.41–0.73)	0.58 (0.4-0.76)
SNAPP≥5	0.66 (0.50–0.81)	0.59 (0.48–0.69)	0.63 (0.52–0.74)	0.62 (0.51–0.73)	0.62 (0.52–0.73)	0.62 (0.51–0.73)	0.63 (0.52–0.73)	0.62 (0.51–0.73)	0.63 (0.52–0.73)	0.63 (0.52–0.73)	0.63 (0.5-0.76)
MoRAL	0.68 (0.48–0.87)	0.66 (0.50–0.78)	0.71 (0.57–0.84)	0.70 (0.57–0.82)	0.71 (0.58–0.83)	0.70 (0.58–0.82)	0.71 (0.57–0.83)	0.71 (0.56–0.83)	0.73 (0.58–0.84)	0.74 (0.59–0.86)	0.68 (0.52-0.82)
MoRAL≥314.8	0.67 (0.52–0.84)	0.58 (0.46–0.68)	0.62 (0.51–0.73)	0.61 (0.50–0.72)	0.62 (0.50–0.72)	0.61 (0.49–0.71)	0.60 (0.49–0.71)	0.60 (0.49–0.70)	0.62 (0.50–0.73)	0.61 (0.49–0.71)	0.63 (0.5-0.76)
R3-AFP	0.70 (0.49–0.86)	0.66 (0.49–0.79)	0.70 (0.57–0.82)	0.69 (0.56–0.82)	0.69 (0.56–0.81)	0.67 (0.53–0.80)	0.68 (0.53–0.81)	0.67 (0.52–0.81)	0.70 (0.55–0.82)	0.70 (0.55–0.84)	0.68 (0.52-0.82)
R3-AFP≥3	0.59 (0.44–0.74)	0.59 (0.45–0.72)	0.61 (0.48–0.73)	0.59 (0.47–0.71)	0.59 (0.46–0.71)	0.58 (0.44–0.70)	0.58 (0.44–0.70)	0.58 (0.45–0.70)	0.59 (0.45–0.72)	0.61 (0.46–0.73)	0.59 (0.46-0.7)

	Dynamic C-index (95%CI)									
**Prediction Models**	**1 Year**	**2 Year**	**3 Year**	**4 Year**	**5 Year**	**6 Year**	**7 Year**	**8 Year**	**9 Year**	**10 Year**
AFP	0.43 (0.26–0.6)	0.41 (0.25–0.57)	0.39 (0.25–0.54)	0.38 (0.25–0.51)	0.39 (0.26–0.53)	0.39 (0.26–0.52)	0.4 (0.27–0.54)	0.42 (0.28–0.55)	0.41 (0.28–0.55)	0.41 (0.28–0.55)
AFP≥200	0.72 (0.47–0.97)	0.7 (0.48–0.93)	0.76 (0.57–0.95)	0.79 (0.62–0.96)	0.77 (0.59–0.95)	0.81 (0.66–0.96)	0.78 (0.62–0.95)	0.8 (0.64–0.95)	0.79 (0.64–0.95)	0.79 (0.63–0.95)
PIVKA-II	0.64 (0.45–0.83)	0.6 (0.41–0.79)	0.57 (0.39–0.75)	0.59 (0.42–0.75)	0.57 (0.41–0.74)	0.61 (0.45–0.76)	0.6 (0.45–0.76)	0.62 (0.47–0.76)	0.61 (0.47–0.76)	0.62 (0.47–0.76)
PIVKA-II≥400	0.79 (0.57–1)	0.79 (0.59–1)	0.66 (0.41–0.92)	0.67 (0.43–0.91)	0.65 (0.4–0.89)	0.71 (0.5–0.92)	0.71 (0.5–0.92)	0.73 (0.53–0.92)	0.68 (0.47–0.9)	0.68 (0.47–0.89)
PIVKA-II+AFP	0.62 (0.41–0.82)	0.61 (0.42–0.8)	0.57 (0.39–0.75)	0.57 (0.4–0.74)	0.56 (0.38–0.73)	0.58 (0.41–0.74)	0.57 (0.41–0.74)	0.58 (0.42–0.74)	0.58 (0.42–0.73)	0.58 (0.43–0.74)
PIVKA-II≥400+AFP≥200	0.75 (0.55–0.95)	0.73 (0.56–0.9)	0.72 (0.56–0.88)	0.74 (0.59–0.89)	0.72 (0.57–0.88)	0.77 (0.64–0.9)	0.75 (0.62–0.89)	0.77 (0.64–0.9)	0.75 (0.62–0.89)	0.75 (0.61–0.88)
SNAPP	0.5 (0.32–0.68)	0.53 (0.34–0.71)	0.51 (0.33–0.7)	0.53 (0.35–0.72)	0.53 (0.35–0.71)	0.58 (0.42–0.75)	0.57 (0.4–0.74)	0.58 (0.41–0.75)	0.58 (0.41–0.74)	0.57 (0.41–0.74)
SNAPP≥5	0.63 (0.35–0.91)	0.64 (0.39–0.89)	0.6 (0.34–0.85)	0.65 (0.42–0.88)	0.63 (0.4–0.86)	0.7 (0.5–0.9)	0.69 (0.48–0.89)	0.7 (0.51–0.9)	0.71 (0.52–0.9)	0.71 (0.52–0.9)
MoRAL	0.66 (0.51–0.82)	0.63 (0.46–0.8)	0.6 (0.43–0.76)	0.62 (0.46–0.77)	0.6 (0.45–0.76)	0.64 (0.5–0.78)	0.64 (0.5–0.78)	0.65 (0.51–0.79)	0.64 (0.51–0.78)	0.64 (0.51–0.78)
MoRAL≥314.8	0.8 (0.6–1)	0.82 (0.65–1)	0.74 (0.53–0.95)	0.74 (0.54–0.94)	0.72 (0.51–0.93)	0.77 (0.6–0.95)	0.8 (0.63–0.96)	0.81 (0.65–0.96)	0.76 (0.59–0.94)	0.78 (0.61–0.94)
R3-AFP	0.47 (0.28–0.65)	0.56 (0.37–0.74)	0.54 (0.36–0.72)	0.54 (0.37–0.72)	0.56 (0.39–0.73)	0.63 (0.47–0.78)	0.63 (0.48–0.78)	0.64 (0.49–0.78)	0.63 (0.48–0.78)	0.63 (0.48–0.78)
R3-AFP≥3	0.35 (0.14–0.56)	0.41 (0.16–0.65)	0.42 (0.19–0.66)	0.46 (0.23–0.69)	0.46 (0.24–0.69)	0.57 (0.35–0.79)	0.58 (0.37–0.8)	0.59 (0.37–0.8)	0.58 (0.37–0.79)	0.57 (0.36–0.78)

AUC, area under curve; C-index, concordance index; 95% CI, 95% confidence interval; AFP, alpha-fetoprotein; PIVKA-II, protein induced by vitamin K absence-II; SNAPP, Size and Number, AFP, PIVKA-II, PET; MoRAL, Model for Recurrence After Liver Transplantation; R3-AFP, Recurrence-Risk Reassessment AFP

Supplementary Table 10. Time-dependent AUC, iAUC and C-index of Extra-Hepatic Recurrence Prediction Models of Milan cohort (also shown in Figure 5A, 5C)

	Time-dependent AUC (95% CI)										Integrated AUC (95%CI)
**Predictions Models**	**1 Year**	**2 Year**	**3 Year**	**4 Year**	**5 Year**	**6 Year**	**7 Year**	**8 Year**	**9 Year**	**10 Year**	
AFP	0.56 (0.43–0.69)	0.56 (0.44–0.68)	0.58 (0.46–0.70)	0.57 (0.45–0.68)	0.59 (0.47–0.70)	0.58 (0.47–0.69)	0.57 (0.47–0.69)	0.56 (0.45–0.68)	0.55 (0.43–0.68)	0.53 (0.40–0.63)	0.56 (0.45-0.67)
AFP≥200	0.50 (0.46–0.56)	0.52 (0.47–0.58)	0.53 (0.48–0.60)	0.52 (0.48–0.58)	0.54 (0.49–0.62)	0.54 (0.49–0.61)	0.54 (0.49–0.60)	0.54 (0.48–0.60)	0.54 (0.48–0.60)	0.53 (0.47–0.58)	0.51 (0.47-0.56)
PIVKA-II	0.66 (0.57–0.75)	0.66 (0.56–0.76)	0.68 (0.58–0.77)	0.64 (0.53–0.74)	0.65 (0.55–0.75)	0.65 (0.55–0.75)	0.67 (0.57–0.77)	0.65 (0.56–0.75)	0.66 (0.56–0.75)	0.67 (0.58–0.76)	0.66 (0.57-0.75)
PIVKA-II≥400	0.48 (0.47–0.49)	0.53 (0.48–0.60)	0.54 (0.48–0.62)	0.53 (0.49–0.60)	0.53 (0.48–0.60)	0.53 (0.48–0.60)	0.53 (0.48–0.59)	0.53 (0.48–0.58)	0.52 (0.48–0.58)	0.52 (0.48–0.57)	0.5 (0.48-0.55)
PIVKA-II+AFP	0.60 (0.50–0.70)	0.63 (0.52–0.72)	0.65 (0.56–0.75)	0.61 (0.51–0.72)	0.63 (0.52–0.73)	0.62 (0.52–0.73)	0.62 (0.52–0.72)	0.61 (0.50–0.72)	0.61 (0.49–0.72)	0.59 (0.47–0.69)	0.61 (0.51-0.7)
PIVKA-II≥400+AFP≥200	0.48 (0.45–0.54)	0.53 (0.46–0.61)	0.54 (0.47–0.62)	0.53 (0.47–0.60)	0.54 (0.48–0.62)	0.54 (0.48–0.61)	0.54 (0.49–0.61)	0.54 (0.48–0.61)	0.53 (0.48–0.60)	0.52 (0.46–0.59)	0.5 (0.46-0.57)
SNAPP	0.46 (0.35–0.59)	0.50 (0.38–0.63)	0.53 (0.42–0.64)	0.49 (0.38–0.60)	0.51 (0.40–0.63)	0.51 (0.40–0.62)	0.52 (0.43–0.63)	0.52 (0.42–0.63)	0.52 (0.43–0.62)	0.53 (0.44–0.63)	0.48 (0.37-0.6)
SNAPP≥5	0.50 (0.49–0.50)	0.52 (0.50–0.57)	0.52 (0.50–0.56)	0.52 (0.50–0.56)	0.51 (0.50–0.55)	0.51 (0.50–0.55)	0.51 (0.50–0.54)	0.51 (0.50–0.55)	0.51 (0.50–0.55)	0.51 (0.50–0.54)	0.51 (0.5-0.53)
RETREAT	0.56 (0.40–0.70)	0.58 (0.45–0.70)	0.61 (0.49–0.72)	0.62 (0.51–0.73)	0.63 (0.52–0.73)	0.62 (0.51–0.73)	0.62 (0.52–0.72)	0.62 (0.53–0.73)	0.62 (0.52–0.74)	0.61 (0.50–0.71)	0.58 (0.46-0.71)
RETREAT≥5	0.51 (0.50–0.51)	0.51 (0.50–0.51)	0.51 (0.50–0.51)	0.51 (0.50–0.51)	0.51 (0.50–0.51)	0.51 (0.50–0.51)	0.51 (0.50–0.51)	0.51 (0.50–0.52)	0.51 (0.50–0.52)	0.51 (0.50–0.52)	0.51 (0.5-0.51)
R3-AFP	0.57 (0.45–0.70)	0.60 (0.48–0.72)	0.62 (0.52–0.73)	0.64 (0.53–0.73)	0.65 (0.55–0.75)	0.64 (0.55–0.74)	0.66 (0.56–0.76)	0.66 (0.57–0.75)	0.65 (0.56–0.75)	0.63 (0.49–0.72)	0.6 (0.49-0.72)
R3-AFP≥3	0.51 (0.47–0.57)	0.50 (0.47–0.55)	0.52 (0.48–0.58)	0.52 (0.48–0.57)	0.53 (0.49–0.59)	0.53 (0.48–0.59)	0.53 (0.48–0.59)	0.53 (0.48–0.58)	0.53 (0.48–0.58)	0.53 (0.48–0.57)	0.51 (0.48-0.56)
	Dynamic C-index (95%CI)										
**Prediction Models**	**1 Year**	**2 Year**	**3 Year**	**4 Year**	**5 Year**	**6 Year**	**7 Year**	**8 Year**	**9 Year**	**10 Year**	
AFP	0.44 (0.3–0.58)	0.43 (0.31–0.55)	0.42 (0.31–0.54)	0.46 (0.35–0.58)	0.44 (0.34–0.55)	0.56 (0.47–0.66)	0.59 (0.5–0.69)	0.61 (0.51–0.7)	0.61 (0.52–0.7)	0.61 (0.52–0.71)	
AFP≥200	0.52 (NA–NA)	0.51 (0.28–0.73)	0.49 (0.25–0.73)	0.54 (0.34–0.75)	0.48 (0.24–0.72)	0.55 (0.32–0.78)	0.56 (0.31–0.8)	0.61 (0.38–0.84)	0.67 (0.46–0.89)	0.71 (0.51–0.91)	
PIVKA-II	0.46 (0.34–0.58)	0.44 (0.32–0.56)	0.44 (0.34–0.54)	0.49 (0.39–0.59)	0.45 (0.36–0.55)	0.57 (0.47–0.66)	0.58 (0.48–0.67)	0.63 (0.54–0.72)	0.63 (0.54–0.72)	0.62 (0.53–0.72)	
PIVKA-II≥400	NA (NA–NA)	0.26 (0.24–0.29)	0.35 (0.23–0.47)	0.42 (0.31–0.54)	0.48 (0.38–0.59)	0.76 (0.7–0.81)	0.67 (0.45–0.89)	0.73 (0.54–0.93)	0.78 (0.61–0.95)	0.78 (0.6–0.96)	
PIVKA-II+AFP	0.47 (0.34–0.6)	0.42 (0.31–0.53)	0.4 (0.29–0.51)	0.46 (0.35–0.57)	0.44 (0.33–0.54)	0.58 (0.49–0.68)	0.6 (0.51–0.69)	0.63 (0.55–0.72)	0.63 (0.54–0.72)	0.63 (0.54–0.72)	
PIVKA-II≥400+AFP≥200	0.5 (NA–NA)	0.41 (0.21–0.61)	0.45 (0.27–0.64)	0.51 (0.35–0.67)	0.49 (0.31–0.66)	0.6 (0.41–0.78)	0.55 (0.34–0.76)	0.61 (0.4–0.81)	0.67 (0.48–0.86)	0.69 (0.51–0.88)	
SNAPP	0.41 (0.21–0.61)	0.32 (0.16–0.48)	0.34 (0.19–0.49)	0.42 (0.26–0.58)	0.41 (0.26–0.56)	0.49 (0.35–0.63)	0.46 (0.32–0.6)	0.48 (0.34–0.62)	0.49 (0.34–0.63)	0.49 (0.35–0.64)	
SNAPP≥5	NA (NA–NA)	0.31 (NA–NA)	0.44 (NA–NA)	0.5 (NA–NA)	0.55 (NA–NA)	0.77 (NA–NA)	0.68 (0.31–1)	0.74 (0.42–1)	0.78 (0.5–1)	0.81 (0.56–1)	
RETREAT	0.48 (0.33–0.62)	0.47 (0.35–0.6)	0.47 (0.34–0.6)	0.48 (0.34–0.61)	0.49 (0.36–0.62)	0.64 (0.51–0.77)	0.65 (0.53–0.78)	0.66 (0.53–0.79)	0.67 (0.54–0.79)	0.67 (0.54–0.79)	
RETREAT≥5	NA (NA–NA)	NA (NA–NA)	NA (NA–NA)	NA (NA–NA)	NA (NA–NA)	NA (NA–NA)	NA (NA–NA)	NA (NA–NA)	NA (NA–NA)	NA (NA–NA)	
R3-AFP	0.44 (0.23–0.65)	0.43 (0.25–0.61)	0.46 (0.3–0.63)	0.47 (0.31–0.63)	0.49 (0.33–0.65)	0.65 (0.52–0.78)	0.66 (0.54–0.79)	0.68 (0.55–0.8)	0.7 (0.57–0.82)	0.7 (0.58–0.81)	
R3-AFP≥3	0.6 (0–1)	0.68 (0.09–1)	0.55 (0.04–1)	0.59 (0.12–1)	0.49 (0.06–0.92)	0.74 (0.53–0.96)	0.67 (0.41–0.92)	0.69 (0.45–0.94)	0.71 (0.47–0.95)	0.7 (0.45–0.94)	

AUC, area under curve; C-index, concordance index; 95% CI, 95% confidence interval; AFP, alpha-fetoprotein; PIVKA-II, protein induced by vitamin K absence-II; SNAPP, Size and Number, AFP, PIVKA-II, PET; RETREAT, Risk Estimation of Tumor Recurrence After Transplant; R3-AFP, Recurrence-Risk Reassessment AFP

Supplementary Table 11. Time-dependent AUC, iAUC and C-index of Extra-Hepatic Recurrence Prediction Models of Beyond Milan cohort (also shown in Figure 5B, 5D)

	Time-dependent AUC (95% CI)										Integrated AUC
**Prediction Models**	**1 Year**	**2 Year**	**3 Year**	**4 Year**	**5 Year**	**6 Year**	**7 Year**	**8 Year**	**9 Year**	**10 Year**	**(95%CI)**
AFP	0.76 (0.65–0.86)	0.70 (0.58–0.79)	0.69 (0.56–0.79)	0.62 (0.50–0.72)	0.63 (0.51–0.74)	0.61 (0.48–0.73)	0.61 (0.46–0.71)	0.62 (0.49–0.73)	0.59 (0.42–0.70)	0.59 (0.43–0.69)	0.71 (0.61-.0.8)
AFP≥200	0.69 (0.59–0.80)	0.67 (0.60–0.75)	0.66 (0.59–0.73)	0.64 (0.57–0.71)	0.65 (0.58–0.72)	0.64 (0.56–0.72)	0.65 (0.57–0.72)	0.65 (0.58–0.72)	0.65 (0.56–0.71)	0.66 (0.58–0.72)	0.68 (0.6-0.76)
PIVKA-II	0.72 (0.59–0.84)	0.67 (0.56–0.76)	0.67 (0.56–0.76)	0.67 (0.56–0.76)	0.68 (0.58–0.78)	0.68 (0.58–0.76)	0.68 (0.56–0.77)	0.67 (0.55–0.76)	0.68 (0.55–0.78)	0.69 (0.56–0.78)	0.7 (0.59-0.79)
PIVKA-II≥400	0.68 (0.58–0.79)	0.61 (0.52–0.69)	0.62 (0.54–0.70)	0.62 (0.54–0.69)	0.63 (0.55–0.71)	0.61 (0.54–0.69)	0.61 (0.51–0.68)	0.60 (0.52–0.68)	0.62 (0.53–0.69)	0.62 (0.52–0.70)	0.65 (0.56-0.74)
PIVKA-II+AFP	0.75 (0.63–0.85)	0.71 (0.62–0.79)	0.71 (0.61–0.80)	0.70 (0.60–0.78)	0.71 (0.62–0.80)	0.70 (0.60–0.79)	0.70 (0.58–0.79)	0.69 (0.57–0.78)	0.70 (0.58–0.79)	0.71 (0.60–0.80)	0.73 (0.62-0.92)
PIVKA-II≥400+AFP≥200	0.77 (0.66–0.87)	0.72 (0.62–0.80)	0.72 (0.62–0.80)	0.70 (0.60–0.77)	0.71 (0.62–0.79)	0.69 (0.59–0.76)	0.69 (0.59–0.77)	0.69 (0.58–0.76)	0.70 (0.59–0.77)	0.71 (0.60–0.78)	0.74 (0.64-0.92)
SNAPP	0.81 (0.68–0.91)	0.75 (0.64–0.84)	0.75 (0.65–0.85)	0.73 (0.62–0.82)	0.74 (0.63–0.83)	0.72 (0.62–0.82)	0.73 (0.63–0.81)	0.72 (0.61–0.81)	0.71 (0.58–0.79)	0.72 (0.58–0.81)	0.77 (0.67-0.96)
SNAPP≥5	0.75 (0.65–0.84)	0.72 (0.63–0.80)	0.73 (0.65–0.81)	0.70 (0.61–0.77)	0.71 (0.63–0.78)	0.69 (0.61–0.76)	0.70 (0.61–0.77)	0.69 (0.61–0.77)	0.69 (0.59–0.76)	0.70 (0.61–0.76)	0.73 (0.65-0.9)
MoRAL	0.76 (0.64–0.86)	0.72 (0.63–0.81)	0.73 (0.62–0.81)	0.71 (0.60–0.79)	0.72 (0.62–0.80)	0.71 (0.61–0.80)	0.71 (0.59–0.80)	0.70 (0.59–0.79)	0.71 (0.58–0.79)	0.72 (0.59–0.80)	0.74 (0.64-0.92)
MoRAL≥314.8	0.68 (0.57–0.77)	0.61 (0.53–0.70)	0.61 (0.53–0.69)	0.61 (0.53–0.69)	0.62 (0.55–0.69)	0.61 (0.53–0.68)	0.60 (0.51–0.67)	0.59 (0.51–0.67)	0.61 (0.53–0.67)	0.60 (0.51–0.67)	0.64 (0.55-0.73)
R3-AFP	0.87 (0.80–0.94)	0.79 (0.71–0.86)	0.81 (0.73–0.88)	0.82 (0.74–0.88)	0.81 (0.74–0.88)	0.78 (0.69–0.86)	0.78 (0.67–0.85)	0.77 (0.66–0.85)	0.78 (0.66–0.85)	0.79 (0.67–0.87)	0.83 (0.75-0.89)
R3-AFP≥3	0.74 (0.68–0.80)	0.73 (0.66–0.80)	0.74 (0.67–0.81)	0.74 (0.67–0.81)	0.75 (0.66–0.82)	0.72 (0.62–0.79)	0.70 (0.60–0.78)	0.71 (0.60–0.78)	0.71 (0.59–0.79)	0.72 (0.60–0.80)	0.74 (0.68-0.79)

	Dynamic C-index (95%CI)									
**Predictions Models**	**1 Year**	**2 Year**	**3 Year**	**4 Year**	**5 Year**	**6 Year**	**7 Year**	**8 Year**	**9 Year**	**10 Year**
AFP	0.59 (0.48–0.71)	0.61 (0.52–0.71)	0.65 (0.57–0.73)	0.67 (0.59–0.74)	0.66 (0.58–0.73)	0.66 (0.6–0.73)	0.65 (0.58–0.72)	0.64 (0.57–0.71)	0.64 (0.57–0.71)	0.64 (0.57–0.71)
AFP≥200	0.61 (0.4–0.81)	0.62 (0.46–0.78)	0.69 (0.54–0.83)	0.72 (0.59–0.85)	0.71 (0.57–0.84)	0.76 (0.64–0.87)	0.74 (0.62–0.86)	0.75 (0.64–0.87)	0.75 (0.64–0.87)	0.75 (0.64–0.87)
PIVKA-II	0.59 (0.47–0.7)	0.62 (0.53–0.7)	0.6 (0.52–0.69)	0.6 (0.52–0.69)	0.59 (0.51–0.68)	0.63 (0.55–0.7)	0.62 (0.54–0.7)	0.63 (0.55–0.71)	0.62 (0.55–0.7)	0.62 (0.55–0.7)
PIVKA-II≥400	0.68 (0.48–0.87)	0.77 (0.64–0.9)	0.69 (0.54–0.84)	0.7 (0.56–0.84)	0.68 (0.54–0.83)	0.74 (0.62–0.87)	0.75 (0.63–0.87)	0.76 (0.65–0.88)	0.73 (0.6–0.85)	0.73 (0.6–0.85)
PIVKA-II+AFP	0.6 (0.47–0.72)	0.62 (0.53–0.7)	0.61 (0.52–0.7)	0.61 (0.53–0.7)	0.6 (0.52–0.69)	0.64 (0.57–0.72)	0.64 (0.57–0.72)	0.65 (0.58–0.73)	0.65 (0.57–0.72)	0.65 (0.57–0.72)
PIVKA-II≥400+AFP≥200	0.63 (0.49–0.77)	0.67 (0.56–0.78)	0.68 (0.58–0.77)	0.7 (0.61–0.79)	0.69 (0.59–0.79)	0.75 (0.66–0.83)	0.73 (0.65–0.82)	0.75 (0.66–0.83)	0.74 (0.65–0.82)	0.73 (0.65–0.82)
SNAPP	0.58 (0.44–0.73)	0.67 (0.59–0.76)	0.67 (0.59–0.75)	0.69 (0.61–0.77)	0.69 (0.61–0.77)	0.73 (0.66–0.81)	0.72 (0.65–0.8)	0.73 (0.66–0.81)	0.73 (0.65–0.81)	0.73 (0.65–0.8)
SNAPP≥5	0.64 (0.43–0.85)	0.71 (0.57–0.86)	0.7 (0.57–0.84)	0.75 (0.62–0.87)	0.74 (0.61–0.86)	0.79 (0.69–0.89)	0.78 (0.68–0.89)	0.8 (0.7–0.9)	0.8 (0.71–0.9)	0.8 (0.71–0.9)
MoRAL	0.6 (0.48–0.72)	0.63 (0.54–0.71)	0.62 (0.53–0.7)	0.63 (0.55–0.71)	0.62 (0.54–0.7)	0.66 (0.58–0.73)	0.65 (0.58–0.73)	0.66 (0.59–0.73)	0.66 (0.58–0.73)	0.66 (0.58–0.73)
MoRAL≥314.8	0.64 (0.44–0.84)	0.74 (0.6–0.89)	0.69 (0.53–0.84)	0.69 (0.54–0.84)	0.67 (0.52–0.83)	0.73 (0.6–0.86)	0.76 (0.63–0.88)	0.77 (0.65–0.89)	0.73 (0.6–0.85)	0.74 (0.62–0.87)
R3-AFP	0.51 (0.38–0.64)	0.68 (0.61–0.75)	0.66 (0.57–0.74)	0.66 (0.59–0.74)	0.68 (0.6–0.75)	0.74 (0.67–0.81)	0.74 (0.67–0.81)	0.75 (0.68–0.81)	0.74 (0.68–0.81)	0.74 (0.67–0.81)
R3-AFP≥3	0.87 (0.66–1)	0.78 (0.63–0.93)	0.79 (0.65–0.94)	0.81 (0.67–0.95)	0.81 (0.68–0.95)	0.87 (0.77–0.97)	0.87 (0.78–0.97)	0.87 (0.78–0.96)	0.86 (0.77–0.96)	0.86 (0.76–0.95)

AUC, area under curve; C-index, concordance index; 95% CI, 95% confidence interval; AFP, alpha-fetoprotein; PIVKA-II, protein induced by vitamin K absence-II; SNAPP, Size and Number, AFP, PIVKA-II, PET; MoRAL, Model for Recurrence After Liver Transplantation; R3-AFP, Recurrence-Risk Reassessment AFP

Supplementary Figure 1.

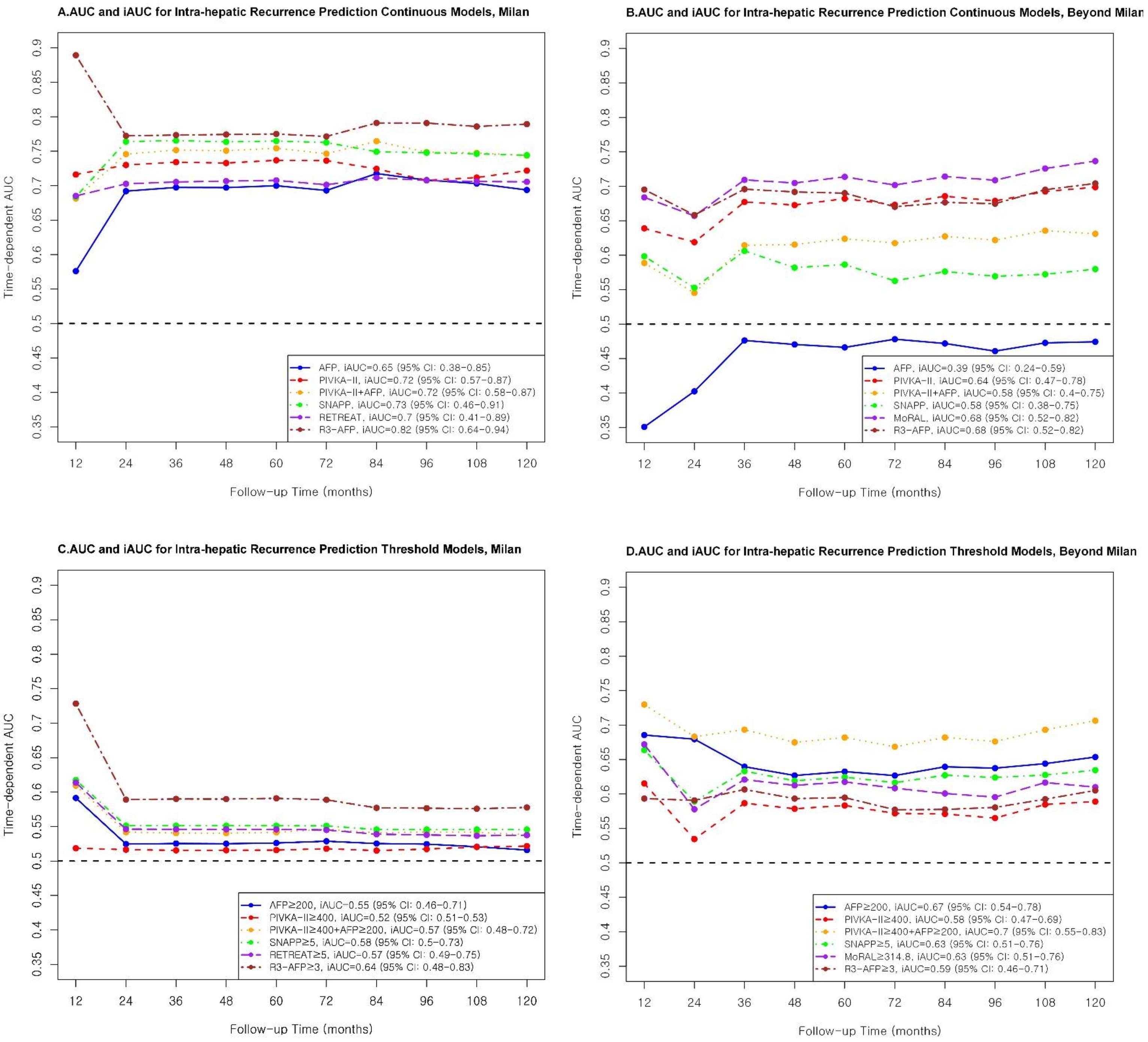

Supplementary Figure 1. AUC and iAUC for intra-hepatic recurrence prediction in Milan and Beyond Milan cohort.

A: Continuous variable–based AUC and iAUC in the Milan cohort.

B: Continuous variable–based AUC and iAUC in the Beyond Milan cohort.

C: Threshold-based AUC and iAUC in the Milan cohort.

D: Threshold-based AUC and iAUC in the Beyond Milan cohort.

Supplementary Figure 2.

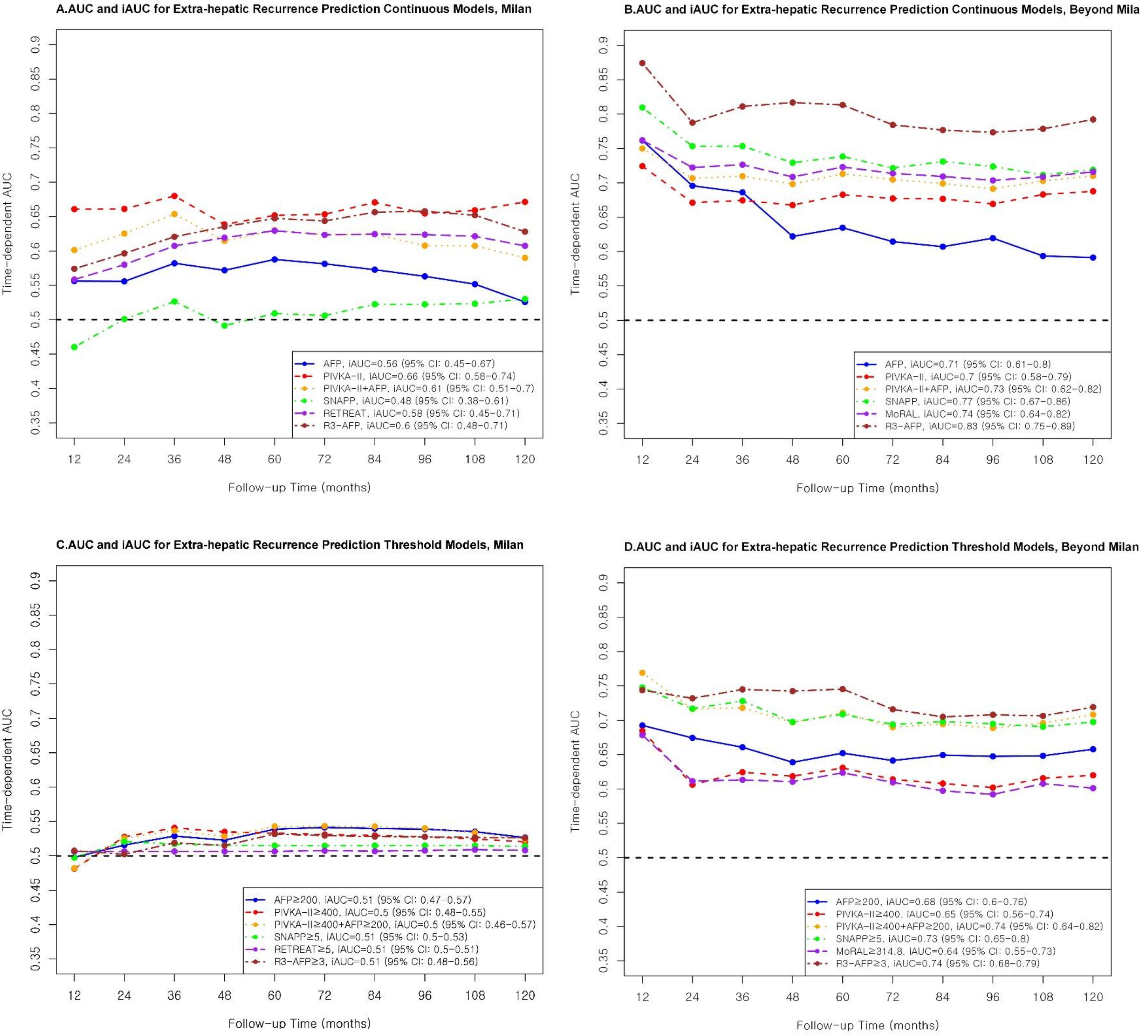

Supplementary Figure 2. AUC and iAUC for extra-hepatic recurrence prediction in Milan and Beyond Milan cohort.

A: Continuous variable–based AUC and iAUC in the Milan cohort.

B: Continuous variable–based AUC and iAUC in the Beyond Milan cohort.

C: Threshold-based AUC and iAUC in the Milan cohort.

D: Threshold-based AUC and iAUC in the Beyond Milan cohort.
